# Supplementary material for: CircParser: a novel streamlined pipeline for circular RNA structure and host gene prediction in non-model organisms
Source: PeerJ. 2020 Mar 16;8:e8757. doi: 10.7717/peerj.8757 (PMC7081776; doi:10.7717/peerj.8757)
Supplement: Table S1 — Number of circular RNAs have been predicted by CircExplorer2, CIRI/ miARma-Seq, CIRI2, find_circ, and circFinder software. The number of common circular RNAs predicted by all methods is also indicated. [file peerj-08-8757-s001.docx]

**Supplementary Table 1**. Sequencing and quality trimming statistics. Number of circular RNAs have been predicted by CircExplorer2, CIRI/ miARma-Seq, CIRI2, find_circ, and circFinder software. The number of common circular RNAs predicted by all methods is also indicated.

| Library name | Number of raw paired-end reads (150 bp) | Number of reads trimmed by quality (Q20) | circExplorer2: Number of identified circRNAs | miARma-Seq: Number of identified circRNAs | CIRI2: Number of identified circRNAs | find_circ: Number of identified circRNAs | circFinder: Number of identified circRNAs | Overlapping circRNAs |
| --- | --- | --- | --- | --- | --- | --- | --- | --- |
| D53 | 38,786,976 | 25,410,262 | 142 | 530 | 808 | 326 | 762 | 45 |
| D63 | 44,498,948 | 35,232,736 | 119 | 394 | 637 | 233 | 660 | 38 |
| D66 | 46,752,428 | 31,107,998 | 144 | 499 | 866 | 376 | 822 | 50 |
| D68 | 35,177,584 | 21,276,704 | 95 | 400 | 594 | 212 | 709 | 27 |
| D70 | 35,260,802 | 21,049,018 | 120 | 452 | 598 | 205 | 631 | 36 |
| D72 | 39,502,652 | 24,933,242 | 120 | 528 | 665 | 249 | 723 | 37 |
| W53 | 37,911,368 | 28,647,182 | 91 | 329 | 515 | 208 | 517 | 20 |
| W63 | 45,608,004 | 29,599,704 | 162 | 541 | 816 | 298 | 881 | 44 |
| W66 | 27,621,420 | 14,100,032 | 155 | 485 | 707 | 230 | 754 | 27 |
| W68 | 46,140,214 | 28,671,324 | 171 | 651 | 869 | 296 | 921 | 53 |
| W70 | 40,783,294 | 25,308,124 | 152 | 634 | 828 | 268 | 928 | 44 |
| W72 | 39,561,872 | 22,819,142 | 99 | 566 | 564 | 180 | 620 | 28 |
