# Supplementary material for: CircParser: a novel streamlined pipeline for circular RNA structure and host gene prediction in non-model organisms
Source: PeerJ. 2020 Mar 16;8:e8757. doi: 10.7717/peerj.8757 (PMC7081776; doi:10.7717/peerj.8757)
Supplement: Table S2 [file peerj-08-8757-s002.docx]

**Supplementary Table 2.** The CircParser pipeline output table for overlapping circRNAs (without structural component analysis)

| Gene ID | Gene coordinates start | Gene coordinates end | Gene of origin for circRNAs ID | Gene of origin for circRNAs | Number of circRNAs | Minimum size, bp | | Maximum size, bp |
| --- | --- | --- | --- | --- | --- | --- | --- | --- |
| NC_031986.2 | 42443745 | 42447945 | XM_019351893.1 | XM_019351893.1 PREDICTED: Oreochromis niloticus SH2 domain-containing adapter protein D (LOC100709134), transcript variant X3, mRNA | 1 | 4231 | 4231 | |
| NC_031986.2 | 3950837 | 3954216 | XM_019351894.1 | XM_019351894.1 PREDICTED: Oreochromis niloticus dystrophin (LOC100694991), mRNA | 1 | 3408 | 3408 | |
| NC_031986.2 | 2525332 | 2526568 | XM_013277247.2 | XM_013277247.2 PREDICTED: Oreochromis niloticus dystrophin (LOC100700501), transcript variant X4, mRNA | 1 | 1265 | 1265 | |
| NC_031985.2 | 23901713 | 23902275 | XM_014335584.1 | XM_014335584.1 PREDICTED: Haplochromis burtoni sorting nexin 13 (snx13), transcript variant X2, mRNA | 1 | 593 | 593 | |
| NC_031984.2 | 15224199 | 15225087 | XM_014336629.1 | XM_014336629.1 PREDICTED: Haplochromis burtoni glutamate receptor-interacting protein 2-like (LOC102303775), transcript variant X6, mRNA | 1 | 919 | 919 | |
| NC_031983.2 | 30813628 | 30814989 | XM_005942849.2 | XM_005942849.2 PREDICTED: Haplochromis burtoni islet cell autoantigen 1, 69kDa (ica1), mRNA | 1 | 1392 | 1392 | |
| NC_031983.2 | 22346989 | 22348856 | XM_019348631.1 | XM_019348631.1 PREDICTED: Oreochromis niloticus solute carrier family 8 member A3 (slc8a3), transcript variant X3, mRNA | 1 | 1898 | 1898 | |
| NC_031982.2 | 16980759 | 16986758 | XM_019347961.1 | XM_019347961.1 PREDICTED: Oreochromis niloticus zinc finger protein 521 (znf521), mRNA | 1 | 6030 | 6030 | |
| NC_031982.2 | 15091152 | 15106042 | XM_005476264.3 | XM_005476264.3 PREDICTED: Oreochromis niloticus glypican-5 (LOC102077503), transcript variant X2, mRNA | 1 | 14921 | 14921 | |
| NC_031981.2 | 37978522 | 37981100 | XM_019346667.1 | XM_019346667.1 PREDICTED: Oreochromis niloticus SET binding factor 1 (sbf1), transcript variant X4, mRNA | 1 | 2609 | 2609 | |
| NC_031981.2 | 32616550 | 32617575 | XM_003452353.4 | XM_003452353.4 PREDICTED: Oreochromis niloticus synapse defective Rho GTPase homolog 2 (syde2), mRNA | 1 | 1056 | 1056 | |
| NC_031981.2 | 27267347 | 27267889 | XM_003457295.4 | XM_003457295.4 PREDICTED: Oreochromis niloticus GDP-mannose 4,6-dehydratase (gmds), mRNA | 1 | 573 | 573 | |
| NC_031981.2 | 10272055 | 10281829 | XR_001224370.2 | XR_001224370.2 PREDICTED: Oreochromis niloticus ELKS/Rab6-interacting/CAST family member 1 (LOC100690550), transcript variant X10, misc_RNA | 1 | 9805 | 9805 | |
| NC_031980.2 | 896114 | 897054 | XR_002058847.1 | XR_002058847.1 PREDICTED: Oreochromis niloticus CDC42 binding protein kinase alpha (cdc42bpa), transcript variant X2, misc_RNA | 1 | 967 | 967 | |
| NC_031980.2 | 32787027 | 32789391 | XM_019346232.1 | XM_019346232.1 PREDICTED: Oreochromis niloticus transmembrane protein 38A (tmem38a), transcript variant X2, mRNA | 1 | 2395 | 2395 | |
| NC_031979.2 | 3312024 | 3324963 | XM_019367617.1 | XM_019367617.1 PREDICTED: Oreochromis niloticus BCAS3, microtubule associated cell migration factor (bcas3), transcript variant X9, mRNA | 1 | 12968 | 12968 | |
| NC_031979.2 | 23188464 | 23189463 | XM_005459369.3 | XM_005459369.3 PREDICTED: Oreochromis niloticus cut like homeobox 1 (cux1), transcript variant X2, mRNA | 1 | 1030 | 1030 | |
| NC_031978.2 | 97182 | 105225 | XM_006809945.1 | XM_006809945.1 PREDICTED: Neolamprologus brichardi LIM domain-binding protein 3-like (LOC102797234), mRNA | 1 | 8069 | 8069 | |
| NC_031978.2 | 30702661 | 30704116 | XM_005461429.3 | XM_005461429.3 PREDICTED: Oreochromis niloticus piezo type mechanosensitive ion channel component 1 (piezo1), mRNA | 1 | 1486 | 1486 | |
| NC_031976.2 | 95036 | 95722 | XR_002059667.1 | XR_002059667.1 PREDICTED: Oreochromis niloticus centrosomal protein 162 (cep162), transcript variant X11, misc_RNA | 1 | 711 | 711 | |
| NC_031976.2 | 4608763 | 4618143 | XM_019358987.1 | XM_019358987.1 PREDICTED: Oreochromis niloticus RNA-directed DNA polymerase from mobile element jockey-like (LOC109202218), partial mRNA | 1 | 9409 | 9409 | |
| NC_031976.2 | 36016595 | 36018688 | XM_013275037.2 | XM_013275037.2 PREDICTED: Oreochromis niloticus lamin A/C (lmna), transcript variant X2, mRNA | 1 | 2124 | 2124 | |
| NC_031973.2 | 9903733 | 9905025 | XM_019362446.1 | XM_019362446.1 PREDICTED: Oreochromis niloticus eukaryotic elongation factor 2 kinase (eef2k), transcript variant X7, mRNA | 1 | 1321 | 1321 | |
| NC_031973.2 | 6541346 | 6542303 | XM_019362294.1 | XM_019362294.1 PREDICTED: Oreochromis niloticus ankyrin-3 (LOC100703272), transcript variant X11, mRNA | 1 | 986 | 986 | |
| NC_031973.2 | 4664930 | 4672518 | XM_019362334.1 | XM_019362334.1 PREDICTED: Oreochromis niloticus microtubule-associated protein tau (LOC100696807), transcript variant X17, mRNA | 1 | 7617 | 7617 | |
| NC_031973.2 | 25289302 | 25292744 | XM_019362085.1 | XM_019362085.1 PREDICTED: Oreochromis niloticus C-Jun-amino-terminal kinase-interacting protein 4 (LOC100690161), transcript variant X3, mRNA | 1 | 3473 | 3473 | |
| NC_031973.2 | 1854460 | 1854640 | XM_006793296.1 | NOT ASSIGNED | 1 | 209 | 209 | |
| NC_031972.2 | 9033630 | 9035959 | XM_019360763.1 | XM_019360763.1 PREDICTED: Oreochromis niloticus myocyte-specific enhancer factor 2C (LOC100706088), transcript variant X4, mRNA | 1 | 2358 | 2358 | |
| NC_031972.2 | 61245143 | 61245486 | XM_019361891.1 | XM_019361891.1 PREDICTED: Oreochromis niloticus microtubule associated monooxygenase, calponin and LIM domain containing 3 (mical3), transcript variant X3, mRNA | 1 | 374 | 374 | |
| NC_031972.2 | 52750455 | 52750889 | XM_019361621.1 | XM_019361621.1 PREDICTED: Oreochromis niloticus tropomyosin 1 (alpha) (tpm1), transcript variant X11, mRNA | 1 | 465 | 465 | |
| NC_031972.2 | 48409310 | 48413963 | XM_014335746.1 | XM_014335746.1 PREDICTED: Haplochromis burtoni CUGBP Elav-like family member 2 (LOC102310863), mRNA | 1 | 4684 | 4684 | |
| NC_031972.2 | 41189270 | 41189586 | XM_019357934.1 | XM_019357934.1 PREDICTED: Oreochromis niloticus suppression of tumorigenicity 7 (st7), transcript variant X1, mRNA | 1 | 347 | 347 | |
| NC_031972.2 | 3583995 | 3590213 | AB067589.1 | AB067589.1 Melanochromis joanjohnsonae DNA, SINE sequence, locus:1613 | 1 | 6247 | 6247 | |
| NC_031972.2 | 3497910 | 3499132 | XM_003439390.3 | XM_003439390.3 PREDICTED: Oreochromis niloticus troponin I, cardiac muscle (LOC100706619), mRNA | 1 | 1251 | 1251 | |
| NC_031972.2 | 32935904 | 32936219 | XM_006782086.1 | NOT ASSIGNED | 1 | 346 | 346 | |
| NC_031971.2 | 42021773 | 42028544 | AB270897.1 | AB270897.1 Oreochromis niloticus MHC class IA antigen UBA1, UBA2, UAA1 genes, partial cds, UAA3 and UAA2 pseudogenes, UAA4, UAA5 and UAA6 pseudogene fragments | 1 | 6802 | 6802 | |
| NC_031971.2 | 4075465 | 4076159 | XM_005461334.3 | XM_005461334.3 PREDICTED: Oreochromis niloticus endoplasmic reticulum oxidoreductase 1 beta (ero1b), transcript variant X2, mRNA | 1 | 723 | 723 | |
| NC_031971.2 | 30305630 | 30307437 | XM_019359755.1 | XM_019359755.1 PREDICTED: Oreochromis niloticus gamma-adducin (LOC100699311), transcript variant X7, mRNA | 1 | 1838 | 1838 | |
| NC_031971.2 | 27059186 | 27061091 | XM_006787490.1 | XM_006787490.1 PREDICTED: Neolamprologus brichardi ubinuclein-2-like (LOC102799674), mRNA | 1 | 1936 | 1936 | |
| NC_031970.2 | 24263108 | 24264783 | XM_019358977.1 | XM_019358977.1 PREDICTED: Oreochromis niloticus gastrula zinc finger protein XlCGF57.1 (LOC100709046), transcript variant X26, mRNA | 1 | 1706 | 1706 | |
| NC_031967.2 | 17263036 | 17268992 | XM_003458877.4 | XM_003458877.4 PREDICTED: Oreochromis niloticus acyl-CoA dehydrogenase, very long chain (acadvl), mRNA | 1 | 5987 | 5987 | |
| NC_031967.2 | 10565666 | 10581723 | XM_019348213.1 | XM_019348213.1 PREDICTED: Oreochromis niloticus pyruvate carboxylase (pc), transcript variant X2, mRNA | 1 | 16088 | 16088 | |
| NC_031966.2 | 29940845 | 29941924 | XM_005467991.3 | XM_005467991.3 PREDICTED: Oreochromis niloticus SH3 domain and tetratricopeptide repeats 2 (sh3tc2), transcript variant X2, mRNA | 1 | 1110 | 1110 | |
| NC_031966.2 | 17389009 | 17402230 | XM_019364388.1 | XM_019364388.1 PREDICTED: Oreochromis niloticus multidrug resistance-associated protein 5 (LOC100710139), transcript variant X2, mRNA | 1 | 13252 | 13252 | |
| NC_031965.2 | 37435820 | 37436209 | XM_012920517.1 | XM_012920517.1 PREDICTED: Maylandia zebra puratrophin-1-like (LOC101472683), transcript variant X2, mRNA | 1 | 420 | 420 | |
| Gene ID | Gene coordinates start | Gene coordinates end | Gene of origin for circRNAs ID | Gene of origin for circRNAs | Number of circRNAs | Minimum size, bp | Maximum size, bp | |
| NC_031987.2 | 29766835 | 29768505 | XM_019353092.1 | XM_019353092.1 PREDICTED: Oreochromis niloticus leucine rich repeats and calponin homology domain containing 1 (lrch1), transcript variant X5, mRNA | 1 | 1701 | 1701 | |
| NC_031986.2 | 45119249 | 45119970 | XM_013268343.2 | XM_013268343.2 PREDICTED: Oreochromis niloticus nexilin F-actin binding protein (nexn), transcript variant X3, mRNA | 1 | 752 | 752 | |
| NC_031986.2 | 35080413 | 35089898 | XM_019351928.1 | XM_019351928.1 PREDICTED: Oreochromis niloticus semaphorin 6B (sema6b), transcript variant X5, mRNA | 1 | 9516 | 9516 | |
| NC_031986.2 | 11263255 | 11266860 | XM_019351651.1 | XM_019351651.1 PREDICTED: Oreochromis niloticus muscleblind-like protein 2a (LOC100695201), transcript variant X5, mRNA | 1 | 3636 | 3636 | |
| NC_031984.2 | 4519550 | 4524223 | XM_019349722.1 | XM_019349722.1 PREDICTED: Oreochromis niloticus solute carrier family 6 member 8 (slc6a8), transcript variant X3, mRNA | 1 | 4702 | 4702 | |
| NC_031984.2 | 22151177 | 22155328 | XM_019349850.1 | XM_019349850.1 PREDICTED: Oreochromis niloticus kinesin family member 1B (kif1b), transcript variant X7, mRNA | 1 | 4182 | 4182 | |
| NC_031983.2 | 27428208 | 27430808 | XM_019348906.1 | XM_019348906.1 PREDICTED: Oreochromis niloticus runt related transcription factor 3 (runx3), transcript variant X3, mRNA | 1 | 2631 | 2631 | |
| NC_031982.2 | 4477984 | 4478859 | XM_019348368.1 | XM_019348368.1 PREDICTED: Oreochromis niloticus mitochondrial ribosomal protein L37 (mrpl37), transcript variant X3, mRNA | 1 | 904 | 904 | |
| NC_031982.2 | 34117602 | 34118973 | XM_005464201.3 | XM_005464201.3 PREDICTED: Oreochromis niloticus ras GTPase-activating protein nGAP (LOC100701601), mRNA | 1 | 1402 | 1402 | |
| NC_031982.2 | 1584077 | 1587668 | XR_267431.3 | XR_267431.3 PREDICTED: Oreochromis niloticus junctophilin-1 (LOC100706964), transcript variant X2, misc_RNA | 1 | 3620 | 3620 | |
| NC_031982.2 | 15053320 | 15106042 | XM_005476264.3 | XM_005476264.3 PREDICTED: Oreochromis niloticus glypican-5 (LOC102077503), transcript variant X2, mRNA | 1 | 52753 | 52753 | |
| NC_031981.2 | 9980440 | 9981823 | XM_006781144.1 | XM_006781144.1 PREDICTED: Neolamprologus brichardi round spermatid basic protein 1-like protein-like (LOC102777401), mRNA | 1 | 1412 | 1412 | |
| NC_031981.2 | 27267347 | 27267889 | XM_003457295.4 | XM_003457295.4 PREDICTED: Oreochromis niloticus GDP-mannose 4,6-dehydratase (gmds), mRNA | 1 | 573 | 573 | |
| NC_031981.2 | 10272056 | 10281829 | XR_001224370.2 | XR_001224370.2 PREDICTED: Oreochromis niloticus ELKS/Rab6-interacting/CAST family member 1 (LOC100690550), transcript variant X10, misc_RNA | 1 | 9804 | 9804 | |
| NC_031980.2 | 896114 | 897054 | XR_002058847.1 | XR_002058847.1 PREDICTED: Oreochromis niloticus CDC42 binding protein kinase alpha (cdc42bpa), transcript variant X2, misc_RNA | 1 | 967 | 967 | |
| NC_031980.2 | 32787027 | 32789391 | XM_019346232.1 | XM_019346232.1 PREDICTED: Oreochromis niloticus transmembrane protein 38A (tmem38a), transcript variant X2, mRNA | 1 | 2395 | 2395 | |
| NC_031980.2 | 31400423 | 31430226 | XM_004566253.1 | XM_004566253.1 PREDICTED: Maylandia zebra enabled homolog (Drosophila) (enah), transcript variant X7, mRNA | 1 | 29834 | 29834 | |
| NC_031979.2 | 35013862 | 35016025 | XM_013913810.1 | XM_013913810.1 PREDICTED: Pundamilia nyererei hypoxia-inducible factor 1-alpha-like (LOC102195817), mRNA | 1 | 2194 | 2194 | |
| NC_031976.2 | 1443315 | 1444282 | XR_002063740.1 | XR_002063740.1 PREDICTED: Oreochromis niloticus HECT domain and ankyrin repeat containing E3 ubiquitin protein ligase 1 (hace1), transcript variant X2, misc_RNA | 1 | 996 | 996 | |
| NC_031976.2 | 10373686 | 10376021 | XM_005450575.2 | XM_005450575.2 PREDICTED: Oreochromis niloticus growth factor receptor-bound protein 10 (LOC100703704), transcript variant X5, mRNA | 1 | 2366 | 2366 | |
| NC_031975.2 | 6849935 | 6869489 | XM_019354380.1 | XM_019354380.1 PREDICTED: Oreochromis niloticus TSPO associated protein 1 (tspoap1), mRNA | 1 | 19583 | 19583 | |
| NC_031973.2 | 9903733 | 9905025 | XM_019362446.1 | XM_019362446.1 PREDICTED: Oreochromis niloticus eukaryotic elongation factor 2 kinase (eef2k), transcript variant X7, mRNA | 1 | 1321 | 1321 | |
| NC_031973.2 | 1854460 | 1854640 | XM_006793296.1 | NOT ASSIGNED | 1 | 209 | 209 | |
| NC_031972.2 | 9033630 | 9035959 | XM_019360763.1 | XM_019360763.1 PREDICTED: Oreochromis niloticus myocyte-specific enhancer factor 2C (LOC100706088), transcript variant X4, mRNA | 1 | 2358 | 2358 | |
| NC_031972.2 | 5643019 | 5644431 | XM_013265378.2 | XM_013265378.2 PREDICTED: Oreochromis niloticus protein-methionine sulfoxide oxidase mical2b (LOC100709311), transcript variant X4, mRNA | 1 | 1441 | 1441 | |
| NC_031972.2 | 48409310 | 48413963 | XM_014335746.1 | XM_014335746.1 PREDICTED: Haplochromis burtoni CUGBP Elav-like family member 2 (LOC102310863), mRNA | 1 | 4684 | 4684 | |
| NC_031972.2 | 38143832 | 38146481 | XM_019361470.1 | NOT ASSIGNED | 1 | 2680 | 2680 | |
| NC_031972.2 | 3583995 | 3584608 | XM_005449241.2 | XM_005449241.2 PREDICTED: Oreochromis niloticus troponin T3, fast skeletal type (tnnt3), transcript variant X6, mRNA | 1 | 642 | 642 | |
| NC_031972.2 | 13315661 | 13321936 | XM_005454468.3 | XM_005454468.3 PREDICTED: Oreochromis niloticus microtubule associated serine/threonine kinase family member 4 (mast4), mRNA | 1 | 6306 | 6306 | |
| NC_031971.2 | 30305630 | 30319707 | AB270897.1 | AB270897.1 Oreochromis niloticus MHC class IA antigen UBA1, UBA2, UAA1 genes, partial cds, UAA3 and UAA2 pseudogenes, UAA4, UAA5 and UAA6 pseudogene fragments | 1 | 14108 | 14108 | |
| NC_031971.2 | 27059186 | 27061091 | XM_006787490.1 | XM_006787490.1 PREDICTED: Neolamprologus brichardi ubinuclein-2-like (LOC102799674), mRNA | 1 | 1936 | 1936 | |
| NC_031971.2 | 24932987 | 24933518 | XM_013272974.2 | XM_013272974.2 PREDICTED: Oreochromis niloticus nuclear factor 1 X-type (LOC100690838), transcript variant X14, mRNA | 1 | 562 | 562 | |
| NC_031970.2 | 32666106 | 32668191 | XM_005450143.3 | XM_005450143.3 PREDICTED: Oreochromis niloticus PDZ domain containing ring finger 3 (pdzrn3), transcript variant X2, mRNA | 1 | 2116 | 2116 | |
| NC_031969.2 | 35390737 | 35394011 | XM_019357502.1 | XM_019357502.1 PREDICTED: Oreochromis niloticus cell division cycle 27 (cdc27), transcript variant X2, mRNA | 1 | 3305 | 3305 | |
| NC_031967.2 | 3188564 | 3191481 | AB270897.1 | AB270897.1 Oreochromis niloticus MHC class IA antigen UBA1, UBA2, UAA1 genes, partial cds, UAA3 and UAA2 pseudogenes, UAA4, UAA5 and UAA6 pseudogene fragments | 1 | 2946 | 2946 | |
| NC_031967.2 | 10565666 | 10581723 | XM_019348213.1 | XM_019348213.1 PREDICTED: Oreochromis niloticus pyruvate carboxylase (pc), transcript variant X2, mRNA | 1 | 16088 | 16088 | |
| NC_031966.2 | 31950713 | 31953718 | XM_013264433.2 | XM_013264433.2 PREDICTED: Oreochromis niloticus zinc finger DHHC-type containing 2 (zdhhc2), transcript variant X3, mRNA | 1 | 3036 | 3036 | |
| NC_031965.2 | 7604686 | 7609175 | XM_019346135.1 | XM_019346135.1 PREDICTED: Oreochromis niloticus neuron navigator 2 (LOC100704374), mRNA | 1 | 4518 | 4518 | |
| Gene ID | Gene coordinates start | Gene coordinates end | Gene of origin for circRNAs ID | Gene of origin for circRNAs | Number of circRNAs | Minimum size, bp | Maximum size, bp | |
| NC_031986.2 | 45119249 | 45119970 | XM_013268343.2 | XM_013268343.2 PREDICTED: Oreochromis niloticus nexilin F-actin binding protein (nexn), transcript variant X3, mRNA | 1 | 752 | 752 | |
| NC_031986.2 | 3950837 | 3954216 | XM_019351894.1 | XM_019351894.1 PREDICTED: Oreochromis niloticus dystrophin (LOC100694991), mRNA | 1 | 3408 | 3408 | |
| NC_031986.2 | 28074347 | 28075775 | XM_019360967.1 | XM_019360967.1 PREDICTED: Oreochromis niloticus peptidylglycine alpha-amidating monooxygenase (pam), transcript variant X2, mRNA | 1 | 1459 | 1459 | |
| NC_031986.2 | 2525332 | 2526568 | XM_013277247.2 | XM_013277247.2 PREDICTED: Oreochromis niloticus dystrophin (LOC100700501), transcript variant X4, mRNA | 1 | 1265 | 1265 | |
| NC_031986.2 | 12928478 | 12928815 | XM_019352179.1 | XM_019352179.1 PREDICTED: Oreochromis niloticus probable uridine nucleosidase 1 (LOC100704461), transcript variant X2, mRNA | 1 | 368 | 368 | |
| NC_031985.2 | 31509826 | 31513172 | XM_019350664.1 | XM_019350664.1 PREDICTED: Oreochromis niloticus CTD phosphatase subunit 1 (ctdp1), transcript variant X3, mRNA | 1 | 3377 | 3377 | |
| NC_031984.2 | 4519550 | 4524223 | XM_019349722.1 | XM_019349722.1 PREDICTED: Oreochromis niloticus solute carrier family 6 member 8 (slc6a8), transcript variant X3, mRNA | 1 | 4702 | 4702 | |
| NC_031984.2 | 20047715 | 20048618 | XM_019349547.1 | XM_019349547.1 PREDICTED: Oreochromis niloticus calcium voltage-gated channel subunit alpha1 S (cacna1s), transcript variant X1, mRNA | 1 | 934 | 934 | |
| NC_031984.2 | 15224199 | 15225087 | XM_014336629.1 | XM_014336629.1 PREDICTED: Haplochromis burtoni glutamate receptor-interacting protein 2-like (LOC102303775), transcript variant X6, mRNA | 1 | 919 | 919 | |
| NC_031982.2 | 4981461 | 4985691 | XM_005464960.3 | XM_005464960.3 PREDICTED: Oreochromis niloticus amylo-alpha-1, 6-glucosidase, 4-alpha-glucanotransferase (agl), transcript variant X2, mRNA | 1 | 4259 | 4259 | |
| NC_031982.2 | 34113913 | 34127018 | XM_005464201.3 | XM_005464201.3 PREDICTED: Oreochromis niloticus ras GTPase-activating protein nGAP (LOC100701601), mRNA | 1 | 13136 | 13136 | |
| NC_031982.2 | 28721832 | 28740958 | XM_019348080.1 | XM_019348080.1 PREDICTED: Oreochromis niloticus partitioning defective 3 homolog (LOC100698856), transcript variant X12, mRNA | 1 | 19157 | 19157 | |
| NC_031982.2 | 27199427 | 27203702 | XM_019348045.1 | XM_019348045.1 PREDICTED: Oreochromis niloticus supervillin (LOC100703985), transcript variant X11, mRNA | 1 | 4306 | 4306 | |
| NC_031982.2 | 22933984 | 22934532 | XM_005476595.3 | XM_005476595.3 PREDICTED: Oreochromis niloticus insulin receptor (LOC100696191), mRNA | 1 | 579 | 579 | |
| NC_031982.2 | 1584077 | 1587668 | XR_267431.3 | XR_267431.3 PREDICTED: Oreochromis niloticus junctophilin-1 (LOC100706964), transcript variant X2, misc_RNA | 1 | 3620 | 3620 | |
| NC_031982.2 | 15091152 | 15093159 | XM_005476264.3 | XM_005476264.3 PREDICTED: Oreochromis niloticus glypican-5 (LOC102077503), transcript variant X2, mRNA | 1 | 2038 | 2038 | |
| NC_031981.2 | 37978522 | 37981100 | XM_019346667.1 | XM_019346667.1 PREDICTED: Oreochromis niloticus SET binding factor 1 (sbf1), transcript variant X4, mRNA | 1 | 2609 | 2609 | |
| NC_031981.2 | 27267347 | 27267889 | XM_003457295.4 | XM_003457295.4 PREDICTED: Oreochromis niloticus GDP-mannose 4,6-dehydratase (gmds), mRNA | 1 | 573 | 573 | |
| NC_031981.2 | 12558116 | 12562115 | XM_019347073.1 | XM_019347073.1 PREDICTED: Oreochromis niloticus voltage-dependent calcium channel subunit alpha-2/delta-1 (LOC100705144), transcript variant X5, mRNA | 1 | 4030 | 4030 | |
| NC_031981.2 | 12545456 | 12549945 | XM_019347073.1 | XM_019347073.1 PREDICTED: Oreochromis niloticus voltage-dependent calcium channel subunit alpha-2/delta-1 (LOC100705144), transcript variant X5, mRNA | 1 | 4520 | 4520 | |
| NC_031979.2 | 573792 | 574347 | XM_019345377.1 | XM_019345377.1 PREDICTED: Oreochromis niloticus A disintegrin and metalloproteinase with thrombospondin motifs 15 (LOC100697734), transcript variant X2, mRNA | 1 | 582 | 582 | |
| NC_031979.2 | 36408021 | 36408711 | XM_012915678.2 | XM_012915678.2 PREDICTED: Maylandia zebra actinin, alpha 4 (actn4), transcript variant X7, mRNA | 1 | 721 | 721 | |
| NC_031979.2 | 24606047 | 24609702 | XM_005459298.3 | XM_005459298.3 PREDICTED: Oreochromis niloticus membrane metalloendopeptidase (mme), transcript variant X4, mRNA | 1 | 3686 | 3686 | |
| NC_031979.2 | 23188464 | 23189463 | XM_005459369.3 | XM_005459369.3 PREDICTED: Oreochromis niloticus cut like homeobox 1 (cux1), transcript variant X2, mRNA | 1 | 1030 | 1030 | |
| NC_031978.2 | 30702661 | 30704116 | XM_005461429.3 | XM_005461429.3 PREDICTED: Oreochromis niloticus piezo type mechanosensitive ion channel component 1 (piezo1), mRNA | 1 | 1486 | 1486 | |
| NC_031978.2 | 26301236 | 26302988 | XM_019366835.1 | XM_019366835.1 PREDICTED: Oreochromis niloticus cytoplasmic polyadenylation element binding protein 3 (cpeb3), transcript variant X7, mRNA | 1 | 1783 | 1783 | |
| NC_031978.2 | 102602 | 105225 | AB270897.1 | AB270897.1 Oreochromis niloticus MHC class IA antigen UBA1, UBA2, UAA1 genes, partial cds, UAA3 and UAA2 pseudogenes, UAA4, UAA5 and UAA6 pseudogene fragments | 1 | 2650 | 2650 | |
| NC_031977.2 | 9580794 | 9583406 | XM_005473041.3 | XM_005473041.3 PREDICTED: Oreochromis niloticus zinc finger DHHC-type containing 8 (zdhhc8), transcript variant X1, mRNA | 1 | 2641 | 2641 | |
| NC_031977.2 | 30892680 | 30896142 | XM_005952579.2 | XM_005952579.2 PREDICTED: Haplochromis burtoni calcium/calmodulin-dependent protein kinase type II subunit beta-like (LOC102306429), mRNA | 1 | 3493 | 3493 | |
| NC_031976.2 | 4608763 | 4618143 | XM_019358987.1 | XM_019358987.1 PREDICTED: Oreochromis niloticus RNA-directed DNA polymerase from mobile element jockey-like (LOC109202218), partial mRNA | 1 | 9409 | 9409 | |
| NC_031976.2 | 36016595 | 36018688 | XM_013275037.2 | XM_013275037.2 PREDICTED: Oreochromis niloticus lamin A/C (lmna), transcript variant X2, mRNA | 1 | 2124 | 2124 | |
| NC_031974.2 | 3245594 | 3246101 | XM_005461865.3 | XM_005461865.3 PREDICTED: Oreochromis niloticus activin receptor type-2B (LOC100691414), mRNA | 1 | 536 | 536 | |
| NC_031974.2 | 29657534 | 29658263 | XM_019362958.1 | XM_019362958.1 PREDICTED: Oreochromis niloticus supervillin (svil), transcript variant X17, mRNA | 1 | 760 | 760 | |
| NC_031974.2 | 2809045 | 2811938 | XM_019354931.1 | XM_019354931.1 PREDICTED: Oreochromis niloticus receptor-type tyrosine-protein phosphatase mu (LOC100711527), transcript variant X8, mRNA | 1 | 2922 | 2922 | |
| NC_031973.2 | 9903733 | 9905025 | XM_019362446.1 | XM_019362446.1 PREDICTED: Oreochromis niloticus eukaryotic elongation factor 2 kinase (eef2k), transcript variant X7, mRNA | 1 | 1321 | 1321 | |
| NC_031973.2 | 9899165 | 9902986 | XM_006791259.1 | XM_006791259.1 PREDICTED: Neolamprologus brichardi eukaryotic elongation factor 2 kinase-like (LOC102800070), mRNA | 1 | 3850 | 3850 | |
| NC_031973.2 | 1854460 | 1854640 | XM_006793296.1 | NOT ASSIGNED | 1 | 209 | 209 | |
| NC_031972.2 | 9033629 | 9035959 | XM_019360763.1 | XM_019360763.1 PREDICTED: Oreochromis niloticus myocyte-specific enhancer factor 2C (LOC100706088), transcript variant X4, mRNA | 1 | 2359 | 2359 | |
| NC_031972.2 | 48409310 | 48413963 | XM_014335746.1 | XM_014335746.1 PREDICTED: Haplochromis burtoni CUGBP Elav-like family member 2 (LOC102310863), mRNA | 1 | 4684 | 4684 | |
| NC_031972.2 | 46433553 | 46440800 | XM_013270549.2 | XM_013270549.2 PREDICTED: Oreochromis niloticus peroxisome proliferator-activated receptor alpha (LOC100709869), transcript variant X2, mRNA | 1 | 7278 | 7278 | |
| NC_031971.2 | 4670550 | 4672713 | AB505452.1 | AB505452.1 Rana rugosa got1 gene for glutamate oxaloacetate transaminase, partial cds | 1 | 2192 | 2192 | |
| NC_031971.2 | 42021773 | 42028544 | AB270897.1 | AB270897.1 Oreochromis niloticus MHC class IA antigen UBA1, UBA2, UAA1 genes, partial cds, UAA3 and UAA2 pseudogenes, UAA4, UAA5 and UAA6 pseudogene fragments | 1 | 6802 | 6802 | |
| NC_031971.2 | 4075465 | 4076159 | XM_005461334.3 | XM_005461334.3 PREDICTED: Oreochromis niloticus endoplasmic reticulum oxidoreductase 1 beta (ero1b), transcript variant X2, mRNA | 1 | 723 | 723 | |
| NC_031971.2 | 27059186 | 27061091 | XM_006787490.1 | XM_006787490.1 PREDICTED: Neolamprologus brichardi ubinuclein-2-like (LOC102799674), mRNA | 1 | 1936 | 1936 | |
| NC_031971.2 | 24932987 | 24933518 | XM_013272974.2 | XM_013272974.2 PREDICTED: Oreochromis niloticus nuclear factor 1 X-type (LOC100690838), transcript variant X14, mRNA | 1 | 562 | 562 | |
| NC_031970.2 | 38048518 | 38050932 | XM_005452249.3 | XM_005452249.3 PREDICTED: Oreochromis niloticus solute carrier family 41 member 1 (slc41a1), transcript variant X3, mRNA | 1 | 2445 | 2445 | |
| NC_031970.2 | 32666106 | 32668191 | XM_005450143.3 | XM_005450143.3 PREDICTED: Oreochromis niloticus PDZ domain containing ring finger 3 (pdzrn3), transcript variant X2, mRNA | 1 | 2116 | 2116 | |
| NC_031967.2 | 3188565 | 3190679 | XM_019349016.1 | XM_019349016.1 PREDICTED: Oreochromis niloticus echinoderm microtubule associated protein like 3 (eml3), transcript variant X2, mRNA | 1 | 2143 | 2143 | |
| NC_031967.2 | 10565666 | 10581723 | XM_019348213.1 | XM_019348213.1 PREDICTED: Oreochromis niloticus pyruvate carboxylase (pc), transcript variant X2, mRNA | 1 | 16088 | 16088 | |
| NC_031965.2 | 3543147 | 3555777 | XM_019349425.1 | XM_019349425.1 PREDICTED: Oreochromis niloticus PTPRF interacting protein alpha 1 (ppfia1), transcript variant X22, mRNA | 1 | 12659 | 12659 | |
| Gene ID | Gene coordinates start | Gene coordinates end | Gene of origin for circRNAs ID | Gene of origin for circRNAs | Number of circRNAs | Minimum size, bp | Maximum size, bp | |
| NC_031987.2 | 21667160 | 21673355 | AB270897.1 | AB270897.1 Oreochromis niloticus MHC class IA antigen UBA1, UBA2, UAA1 genes, partial cds, UAA3 and UAA2 pseudogenes, UAA4, UAA5 and UAA6 pseudogene fragments | 1 | 6226 | 6226 | |
| NC_031987.2 | 14996343 | 15015105 | XM_019353022.1 | XM_019353022.1 PREDICTED: Oreochromis niloticus plakophilin 4 (pkp4), transcript variant X4, mRNA | 1 | 18793 | 18793 | |
| NC_031986.2 | 2525332 | 2526568 | XM_013277247.2 | XM_013277247.2 PREDICTED: Oreochromis niloticus dystrophin (LOC100700501), transcript variant X4, mRNA | 1 | 1265 | 1265 | |
| NC_031984.2 | 4519550 | 4524223 | XM_019349722.1 | XM_019349722.1 PREDICTED: Oreochromis niloticus solute carrier family 6 member 8 (slc6a8), transcript variant X3, mRNA | 1 | 4702 | 4702 | |
| NC_031984.2 | 34388516 | 34389012 | XM_019349810.1 | XM_019349810.1 PREDICTED: Oreochromis niloticus vacuolar protein sorting 13 homolog D (vps13d), transcript variant X3, mRNA | 1 | 527 | 527 | |
| NC_031984.2 | 22151177 | 22155328 | XM_019349850.1 | XM_019349850.1 PREDICTED: Oreochromis niloticus kinesin family member 1B (kif1b), transcript variant X7, mRNA | 1 | 4182 | 4182 | |
| NC_031984.2 | 22067641 | 22068248 | XM_005478204.3 | XM_005478204.3 PREDICTED: Oreochromis niloticus calsyntenin 1 (clstn1), transcript variant X3, mRNA | 1 | 638 | 638 | |
| NC_031984.2 | 15224199 | 15225087 | XM_014336629.1 | XM_014336629.1 PREDICTED: Haplochromis burtoni glutamate receptor-interacting protein 2-like (LOC102303775), transcript variant X6, mRNA | 1 | 919 | 919 | |
| NC_031982.2 | 4535430 | 4539958 | XM_005464528.2 | XM_005464528.2 PREDICTED: Oreochromis niloticus DEP domain containing 5 (depdc5), transcript variant X17, mRNA | 1 | 4557 | 4557 | |
| NC_031982.2 | 27199427 | 27203702 | XM_019348045.1 | XM_019348045.1 PREDICTED: Oreochromis niloticus supervillin (LOC100703985), transcript variant X11, mRNA | 1 | 4306 | 4306 | |
| NC_031981.2 | 9980440 | 9981823 | XM_006781144.1 | XM_006781144.1 PREDICTED: Neolamprologus brichardi round spermatid basic protein 1-like protein-like (LOC102777401), mRNA | 1 | 1412 | 1412 | |
| NC_031981.2 | 12545456 | 12549945 | XM_019347073.1 | XM_019347073.1 PREDICTED: Oreochromis niloticus voltage-dependent calcium channel subunit alpha-2/delta-1 (LOC100705144), transcript variant X5, mRNA | 1 | 4520 | 4520 | |
| NC_031981.2 | 10272056 | 10281829 | XR_001224370.2 | XR_001224370.2 PREDICTED: Oreochromis niloticus ELKS/Rab6-interacting/CAST family member 1 (LOC100690550), transcript variant X10, misc_RNA | 1 | 9804 | 9804 | |
| NC_031980.2 | 896114 | 897054 | XR_002058847.1 | XR_002058847.1 PREDICTED: Oreochromis niloticus CDC42 binding protein kinase alpha (cdc42bpa), transcript variant X2, misc_RNA | 1 | 967 | 967 | |
| NC_031979.2 | 13301603 | 13305188 | XM_014408648.1 | XM_014408648.1 PREDICTED: Maylandia zebra butyrylcholinesterase (bche), transcript variant X3, mRNA | 1 | 3616 | 3616 | |
| NC_031978.2 | 30702661 | 30704116 | XM_005461429.3 | XM_005461429.3 PREDICTED: Oreochromis niloticus piezo type mechanosensitive ion channel component 1 (piezo1), mRNA | 1 | 1486 | 1486 | |
| NC_031978.2 | 102602 | 105225 | AB270897.1 | AB270897.1 Oreochromis niloticus MHC class IA antigen UBA1, UBA2, UAA1 genes, partial cds, UAA3 and UAA2 pseudogenes, UAA4, UAA5 and UAA6 pseudogene fragments | 1 | 2650 | 2650 | |
| NC_031978.2 | 10245923 | 10250619 | AJ889574.1 | AJ889574.1 Oreochromis mossambicus partial vtg gene for vitellogenin, promoter and exon 1 | 1 | 4727 | 4727 | |
| NC_031976.2 | 36016595 | 36018688 | XM_013275037.2 | XM_013275037.2 PREDICTED: Oreochromis niloticus lamin A/C (lmna), transcript variant X2, mRNA | 1 | 2124 | 2124 | |
| NC_031976.2 | 1773467 | 1774455 | XM_013268385.2 | XM_013268385.2 PREDICTED: Oreochromis niloticus phosphatidylinositide phosphatase SAC1-A (LOC100707989), transcript variant X3, mRNA | 1 | 1017 | 1017 | |
| NC_031973.2 | 9903733 | 9905025 | XM_019362446.1 | XM_019362446.1 PREDICTED: Oreochromis niloticus eukaryotic elongation factor 2 kinase (eef2k), transcript variant X7, mRNA | 1 | 1321 | 1321 | |
| NC_031973.2 | 1854460 | 1854640 | XM_006793296.1 | NOT ASSIGNED | 1 | 209 | 209 | |
| NC_031972.2 | 9033630 | 9035959 | XM_019360763.1 | XM_019360763.1 PREDICTED: Oreochromis niloticus myocyte-specific enhancer factor 2C (LOC100706088), transcript variant X4, mRNA | 1 | 2358 | 2358 | |
| NC_031972.2 | 48409310 | 48413963 | XM_014335746.1 | XM_014335746.1 PREDICTED: Haplochromis burtoni CUGBP Elav-like family member 2 (LOC102310863), mRNA | 1 | 4684 | 4684 | |
| NC_031971.2 | 41823946 | 41827966 | AB270897.1 | AB270897.1 Oreochromis niloticus MHC class IA antigen UBA1, UBA2, UAA1 genes, partial cds, UAA3 and UAA2 pseudogenes, UAA4, UAA5 and UAA6 pseudogene fragments | 1 | 4051 | 4051 | |
| NC_031971.2 | 27059186 | 27061091 | XM_006787490.1 | XM_006787490.1 PREDICTED: Neolamprologus brichardi ubinuclein-2-like (LOC102799674), mRNA | 1 | 1936 | 1936 | |
| NC_031965.2 | 3543147 | 3555777 | XM_019349425.1 | XM_019349425.1 PREDICTED: Oreochromis niloticus PTPRF interacting protein alpha 1 (ppfia1), transcript variant X22, mRNA | 1 | 12659 | 12659 | |
| Gene ID | Gene coordinates start | Gene coordinates end | Gene of origin for circRNAs ID | Gene of origin for circRNAs | Number of circRNAs | Minimum size, bp | Maximum size, bp | |
| NC_031987.2 | 33761246 | 33761533 | XM_019352790.1 | XM_019352790.1 PREDICTED: Oreochromis niloticus titin (LOC100702396), mRNA | 1 | 318 | 318 | |
| NC_031987.2 | 26789645 | 26791360 | XM_019352997.1 | XM_019352997.1 PREDICTED: Oreochromis niloticus nebulin (neb), transcript variant X7, mRNA | 1 | 1746 | 1746 | |
| NC_031986.2 | 45119249 | 45119970 | XM_013268343.2 | XM_013268343.2 PREDICTED: Oreochromis niloticus nexilin F-actin binding protein (nexn), transcript variant X3, mRNA | 1 | 752 | 752 | |
| NC_031986.2 | 3950837 | 3954216 | XM_019351894.1 | XM_019351894.1 PREDICTED: Oreochromis niloticus dystrophin (LOC100694991), mRNA | 1 | 3408 | 3408 | |
| NC_031986.2 | 2525332 | 2526568 | XM_013277247.2 | XM_013277247.2 PREDICTED: Oreochromis niloticus dystrophin (LOC100700501), transcript variant X4, mRNA | 1 | 1265 | 1265 | |
| NC_031985.2 | 22257330 | 22261188 | AB270897.1 | AB270897.1 Oreochromis niloticus MHC class IA antigen UBA1, UBA2, UAA1 genes, partial cds, UAA3 and UAA2 pseudogenes, UAA4, UAA5 and UAA6 pseudogene fragments | 1 | 3889 | 3889 | |
| NC_031984.2 | 35582665 | 35589644 | AH013711.2 | AH013711.2 Oreochromis niloticus KLR3 (KLR3) gene, exons 2 through 7; KLR2 pseudogene, partial sequence; KLR1 gene, complete sequence; KLR4 (KLR4) gene, exons 2 through 7; KLR5 (KLR5) gene, exons 1 through 7; KLR6 and KLR7 pseudogenes, complete sequence; KLR9 (KLR10) pseudogene, exons 4 through 7; KLR8 pseudogene, partial sequence; KLR9 (KLR9) gene, exons 1 through 7; C-type lectin (CLECT2)-like protein gene, complete sequence; C-type lectin natural killer cell receptor-like protein gene, exons 1 and 2; and transposon TX1-like ORF2 pseudogene, partial sequence | 1 | 7010 | 7010 | |
| NC_031984.2 | 22067641 | 22068248 | XM_005478204.3 | XM_005478204.3 PREDICTED: Oreochromis niloticus calsyntenin 1 (clstn1), transcript variant X3, mRNA | 1 | 638 | 638 | |
| NC_031983.2 | 26804436 | 26810832 | XM_005477608.3 | XM_005477608.3 PREDICTED: Oreochromis niloticus apoptosis-stimulating of p53 protein 1 (LOC100705495), transcript variant X3, mRNA | 1 | 6427 | 6427 | |
| NC_031982.2 | 4535430 | 4539958 | XM_005464528.2 | XM_005464528.2 PREDICTED: Oreochromis niloticus DEP domain containing 5 (depdc5), transcript variant X17, mRNA | 1 | 4557 | 4557 | |
| NC_031982.2 | 4477984 | 4478859 | XM_019348368.1 | XM_019348368.1 PREDICTED: Oreochromis niloticus mitochondrial ribosomal protein L37 (mrpl37), transcript variant X3, mRNA | 1 | 904 | 904 | |
| NC_031982.2 | 27199427 | 27203702 | XM_019348045.1 | XM_019348045.1 PREDICTED: Oreochromis niloticus supervillin (LOC100703985), transcript variant X11, mRNA | 1 | 4306 | 4306 | |
| NC_031982.2 | 22933984 | 22934532 | XM_005476595.3 | XM_005476595.3 PREDICTED: Oreochromis niloticus insulin receptor (LOC100696191), mRNA | 1 | 579 | 579 | |
| NC_031982.2 | 15053320 | 15106042 | XM_005476264.3 | XM_005476264.3 PREDICTED: Oreochromis niloticus glypican-5 (LOC102077503), transcript variant X2, mRNA | 1 | 52753 | 52753 | |
| NC_031981.2 | 12545456 | 12549945 | XM_019347073.1 | XM_019347073.1 PREDICTED: Oreochromis niloticus voltage-dependent calcium channel subunit alpha-2/delta-1 (LOC100705144), transcript variant X5, mRNA | 1 | 4520 | 4520 | |
| NC_031981.2 | 10272056 | 10281829 | XR_001224370.2 | XR_001224370.2 PREDICTED: Oreochromis niloticus ELKS/Rab6-interacting/CAST family member 1 (LOC100690550), transcript variant X10, misc_RNA | 1 | 9804 | 9804 | |
| NC_031980.2 | 896114 | 897054 | XR_002058847.1 | XR_002058847.1 PREDICTED: Oreochromis niloticus CDC42 binding protein kinase alpha (cdc42bpa), transcript variant X2, misc_RNA | 1 | 967 | 967 | |
| NC_031980.2 | 4274992 | 4284218 | XR_002056607.1 | XR_002056607.1 PREDICTED: Oreochromis niloticus 1-phosphatidylinositol 4,5-bisphosphate phosphodiesterase beta-1 (LOC100698389), transcript variant X4, misc_RNA | 1 | 9255 | 9255 | |
| NC_031980.2 | 32787027 | 32789391 | XM_019346232.1 | XM_019346232.1 PREDICTED: Oreochromis niloticus transmembrane protein 38A (tmem38a), transcript variant X2, mRNA | 1 | 2395 | 2395 | |
| NC_031978.2 | 30702661 | 30704116 | XM_005461429.3 | XM_005461429.3 PREDICTED: Oreochromis niloticus piezo type mechanosensitive ion channel component 1 (piezo1), mRNA | 1 | 1486 | 1486 | |
| NC_031976.2 | 36016595 | 36018688 | XM_013275037.2 | XM_013275037.2 PREDICTED: Oreochromis niloticus lamin A/C (lmna), transcript variant X2, mRNA | 1 | 2124 | 2124 | |
| NC_031976.2 | 22980651 | 22982460 | XM_005455415.3 | XM_005455415.3 PREDICTED: Oreochromis niloticus chloride voltage-gated channel 1 (clcn1), mRNA | 1 | 1840 | 1840 | |
| NC_031974.2 | 17041291 | 17043827 | XM_019363096.1 | XM_019363096.1 PREDICTED: Oreochromis niloticus DLG associated protein 1 (dlgap1), transcript variant X2, mRNA | 1 | 2567 | 2567 | |
| NC_031973.2 | 9899165 | 9905025 | XM_019362446.1 | XM_019362446.1 PREDICTED: Oreochromis niloticus eukaryotic elongation factor 2 kinase (eef2k), transcript variant X7, mRNA | 1 | 5889 | 5889 | |
| NC_031972.2 | 9033630 | 9035959 | XM_019360763.1 | XM_019360763.1 PREDICTED: Oreochromis niloticus myocyte-specific enhancer factor 2C (LOC100706088), transcript variant X4, mRNA | 1 | 2358 | 2358 | |
| NC_031972.2 | 61245143 | 61245486 | XM_019361891.1 | XM_019361891.1 PREDICTED: Oreochromis niloticus microtubule associated monooxygenase, calponin and LIM domain containing 3 (mical3), transcript variant X3, mRNA | 1 | 374 | 374 | |
| NC_031972.2 | 48409310 | 48413963 | XM_014335746.1 | XM_014335746.1 PREDICTED: Haplochromis burtoni CUGBP Elav-like family member 2 (LOC102310863), mRNA | 1 | 4684 | 4684 | |
| NC_031972.2 | 3583995 | 3584608 | XM_005449241.2 | XM_005449241.2 PREDICTED: Oreochromis niloticus troponin T3, fast skeletal type (tnnt3), transcript variant X6, mRNA | 1 | 642 | 642 | |
| NC_031972.2 | 2011461 | 2012365 | XM_005470209.3 | XM_005470209.3 PREDICTED: Oreochromis niloticus zinc finger C3H1-type containing (zfc3h1), mRNA | 1 | 933 | 933 | |
| NC_031971.2 | 41823946 | 41827966 | AB270897.1 | AB270897.1 Oreochromis niloticus MHC class IA antigen UBA1, UBA2, UAA1 genes, partial cds, UAA3 and UAA2 pseudogenes, UAA4, UAA5 and UAA6 pseudogene fragments | 1 | 4051 | 4051 | |
| NC_031971.2 | 4075465 | 4076159 | XM_005461334.3 | XM_005461334.3 PREDICTED: Oreochromis niloticus endoplasmic reticulum oxidoreductase 1 beta (ero1b), transcript variant X2, mRNA | 1 | 723 | 723 | |
| NC_031971.2 | 24932987 | 24933518 | XM_013272974.2 | XM_013272974.2 PREDICTED: Oreochromis niloticus nuclear factor 1 X-type (LOC100690838), transcript variant X14, mRNA | 1 | 562 | 562 | |
| NC_031967.2 | 32532813 | 32533411 | XM_019353059.1 | XM_019353059.1 PREDICTED: Oreochromis niloticus phosphorylase b kinase regulatory subunit alpha, skeletal muscle isoform (LOC100707493), transcript variant X12, mRNA | 1 | 629 | 629 | |
| NC_031966.2 | 36207537 | 36208018 | XM_019346843.1 | XM_019346843.1 PREDICTED: Oreochromis niloticus ArfGAP with RhoGAP domain, ankyrin repeat and PH domain 3 (arap3), transcript variant X3, mRNA | 1 | 512 | 512 | |
| NC_031966.2 | 31950713 | 31953718 | XM_013264433.2 | XM_013264433.2 PREDICTED: Oreochromis niloticus zinc finger DHHC-type containing 2 (zdhhc2), transcript variant X3, mRNA | 1 | 3036 | 3036 | |
| NC_031965.2 | 3549150 | 3555777 | XM_019349425.1 | XM_019349425.1 PREDICTED: Oreochromis niloticus PTPRF interacting protein alpha 1 (ppfia1), transcript variant X22, mRNA | 1 | 6656 | 6656 | |
| Gene ID | Gene coordinates start | Gene coordinates end | Gene of origin for circRNAs ID | Gene of origin for circRNAs | Number of circRNAs | Minimum size, bp | Maximum size, bp | |
| NC_031987.2 | 31995443 | 31999751 | XM_019353242.1 | XM_019353242.1 PREDICTED: Oreochromis niloticus diacylglycerol kinase beta (LOC100698894), transcript variant X3, mRNA | 1 | 4339 | 4339 | |
| NC_031987.2 | 26790951 | 26791360 | XM_019352997.1 | XM_019352997.1 PREDICTED: Oreochromis niloticus nebulin (neb), transcript variant X7, mRNA | 1 | 440 | 440 | |
| NC_031986.2 | 45119249 | 45119970 | XM_013268343.2 | XM_013268343.2 PREDICTED: Oreochromis niloticus nexilin F-actin binding protein (nexn), transcript variant X3, mRNA | 1 | 752 | 752 | |
| NC_031986.2 | 35080413 | 35089898 | XM_019351928.1 | XM_019351928.1 PREDICTED: Oreochromis niloticus semaphorin 6B (sema6b), transcript variant X5, mRNA | 1 | 9516 | 9516 | |
| NC_031986.2 | 1871952 | 1872657 | XM_005459210.3 | XM_005459210.3 PREDICTED: Oreochromis niloticus rho GTPase-activating protein 1 (LOC100697014), transcript variant X2, mRNA | 1 | 734 | 734 | |
| NC_031986.2 | 12928478 | 12928815 | XM_019352179.1 | XM_019352179.1 PREDICTED: Oreochromis niloticus probable uridine nucleosidase 1 (LOC100704461), transcript variant X2, mRNA | 1 | 368 | 368 | |
| NC_031984.2 | 37049258 | 37070704 | KT688694.1 | KT688694.1 Cheilochromis euchilus voucher C. Darrin Hulsey:4812 ultra conserved element locus uce-575 genomic sequence | 1 | 21477 | 21477 | |
| NC_031984.2 | 15224199 | 15225087 | XM_014336629.1 | XM_014336629.1 PREDICTED: Haplochromis burtoni glutamate receptor-interacting protein 2-like (LOC102303775), transcript variant X6, mRNA | 1 | 919 | 919 | |
| NC_031983.2 | 3862804 | 3867692 | XM_013274982.2 | XM_013274982.2 PREDICTED: Oreochromis niloticus spectrin beta, non-erythrocytic 5 (sptbn5), mRNA | 1 | 4917 | 4917 | |
| NC_031982.2 | 22933984 | 22934532 | XM_005476595.3 | XM_005476595.3 PREDICTED: Oreochromis niloticus insulin receptor (LOC100696191), mRNA | 1 | 579 | 579 | |
| NC_031982.2 | 19902650 | 19903912 | XM_019347931.1 | XM_019347931.1 PREDICTED: Oreochromis niloticus epidermal growth factor receptor (egfr), mRNA | 1 | 1293 | 1293 | |
| NC_031982.2 | 15091151 | 15106042 | XM_005476264.3 | XM_005476264.3 PREDICTED: Oreochromis niloticus glypican-5 (LOC102077503), transcript variant X2, mRNA | 1 | 14922 | 14922 | |
| NC_031982.2 | 14453483 | 14455763 | XM_019347703.1 | XM_019347703.1 PREDICTED: Oreochromis niloticus muscleblind-like protein 1 (LOC100712433), transcript variant X21, mRNA | 1 | 2311 | 2311 | |
| NC_031981.2 | 9980440 | 9981823 | XM_006781144.1 | XM_006781144.1 PREDICTED: Neolamprologus brichardi round spermatid basic protein 1-like protein-like (LOC102777401), mRNA | 1 | 1412 | 1412 | |
| NC_031981.2 | 27267347 | 27267889 | XM_003457295.4 | XM_003457295.4 PREDICTED: Oreochromis niloticus GDP-mannose 4,6-dehydratase (gmds), mRNA | 1 | 573 | 573 | |
| NC_031981.2 | 12545456 | 12549945 | XM_019347073.1 | XM_019347073.1 PREDICTED: Oreochromis niloticus voltage-dependent calcium channel subunit alpha-2/delta-1 (LOC100705144), transcript variant X5, mRNA | 1 | 4520 | 4520 | |
| NC_031981.2 | 12526866 | 12528711 | XM_019347073.1 | XM_019347073.1 PREDICTED: Oreochromis niloticus voltage-dependent calcium channel subunit alpha-2/delta-1 (LOC100705144), transcript variant X5, mRNA | 1 | 1876 | 1876 | |
| NC_031981.2 | 11770508 | 11771010 | XM_019346818.1 | XM_019346818.1 PREDICTED: Oreochromis niloticus plasma membrane calcium-transporting ATPase 1 (LOC100695649), transcript variant X4, mRNA | 1 | 533 | 533 | |
| NC_031980.2 | 896114 | 897054 | XR_002058847.1 | XR_002058847.1 PREDICTED: Oreochromis niloticus CDC42 binding protein kinase alpha (cdc42bpa), transcript variant X2, misc_RNA | 1 | 967 | 967 | |
| NC_031979.2 | 24606047 | 24609702 | XM_005459298.3 | XM_005459298.3 PREDICTED: Oreochromis niloticus membrane metalloendopeptidase (mme), transcript variant X4, mRNA | 1 | 3686 | 3686 | |
| NC_031978.2 | 102602 | 105225 | AB270897.1 | AB270897.1 Oreochromis niloticus MHC class IA antigen UBA1, UBA2, UAA1 genes, partial cds, UAA3 and UAA2 pseudogenes, UAA4, UAA5 and UAA6 pseudogene fragments | 1 | 2650 | 2650 | |
| NC_031977.2 | 10164040 | 10167430 | XM_013271579.2 | XM_013271579.2 PREDICTED: Oreochromis niloticus calcineurin binding protein 1 (cabin1), mRNA | 1 | 3421 | 3421 | |
| NC_031976.2 | 36016595 | 36018688 | XM_013275037.2 | XM_013275037.2 PREDICTED: Oreochromis niloticus lamin A/C (lmna), transcript variant X2, mRNA | 1 | 2124 | 2124 | |
| NC_031975.2 | 7212053 | 7212813 | XM_019363959.1 | XM_019363959.1 PREDICTED: Oreochromis niloticus apoptosis inducing factor, mitochondria associated 1 (aifm1), transcript variant X2, mRNA | 1 | 789 | 789 | |
| NC_031975.2 | 1409416 | 1423947 | XM_019363902.1 | XM_019363902.1 PREDICTED: Oreochromis niloticus vacuole membrane protein 1 (LOC100690150), transcript variant X7, mRNA | 1 | 14560 | 14560 | |
| NC_031973.2 | 9897506 | 9905025 | XM_019362446.1 | XM_019362446.1 PREDICTED: Oreochromis niloticus eukaryotic elongation factor 2 kinase (eef2k), transcript variant X7, mRNA | 1 | 7548 | 7548 | |
| NC_031973.2 | 1854460 | 1854640 | XM_006793296.1 | NOT ASSIGNED | 1 | 209 | 209 | |
| NC_031972.2 | 9033630 | 9035959 | XM_019360763.1 | XM_019360763.1 PREDICTED: Oreochromis niloticus myocyte-specific enhancer factor 2C (LOC100706088), transcript variant X4, mRNA | 1 | 2358 | 2358 | |
| NC_031972.2 | 48409310 | 48413963 | XM_014335746.1 | XM_014335746.1 PREDICTED: Haplochromis burtoni CUGBP Elav-like family member 2 (LOC102310863), mRNA | 1 | 4684 | 4684 | |
| NC_031971.2 | 5222007 | 5223906 | XM_019360418.1 | XM_019360418.1 PREDICTED: Oreochromis niloticus exportin-1 (LOC100700222), transcript variant X2, mRNA | 1 | 1928 | 1928 | |
| NC_031971.2 | 42021773 | 42028544 | AB270897.1 | AB270897.1 Oreochromis niloticus MHC class IA antigen UBA1, UBA2, UAA1 genes, partial cds, UAA3 and UAA2 pseudogenes, UAA4, UAA5 and UAA6 pseudogene fragments | 1 | 6802 | 6802 | |
| NC_031971.2 | 4075465 | 4076159 | XM_005461334.3 | XM_005461334.3 PREDICTED: Oreochromis niloticus endoplasmic reticulum oxidoreductase 1 beta (ero1b), transcript variant X2, mRNA | 1 | 723 | 723 | |
| NC_031971.2 | 27059186 | 27061091 | XM_006787490.1 | XM_006787490.1 PREDICTED: Neolamprologus brichardi ubinuclein-2-like (LOC102799674), mRNA | 1 | 1936 | 1936 | |
| NC_031970.2 | 10361748 | 10362827 | XM_019359074.1 | XM_019359074.1 PREDICTED: Oreochromis niloticus membrane-associated guanylate kinase, WW and PDZ domain-containing protein 3 (LOC100702300), transcript variant X2, mRNA | 1 | 1110 | 1110 | |
| NC_031967.2 | 3188565 | 3190679 | XM_019349016.1 | XM_019349016.1 PREDICTED: Oreochromis niloticus echinoderm microtubule associated protein like 3 (eml3), transcript variant X2, mRNA | 1 | 2143 | 2143 | |
| NC_031966.2 | 31950713 | 31953718 | XM_013264433.2 | XM_013264433.2 PREDICTED: Oreochromis niloticus zinc finger DHHC-type containing 2 (zdhhc2), transcript variant X3, mRNA | 1 | 3036 | 3036 | |
| NC_031966.2 | 29940845 | 29941924 | XM_005467991.3 | XM_005467991.3 PREDICTED: Oreochromis niloticus SH3 domain and tetratricopeptide repeats 2 (sh3tc2), transcript variant X2, mRNA | 1 | 1110 | 1110 | |
| Gene ID | Gene coordinates start | Gene coordinates end | Gene of origin for circRNAs ID | Gene of origin for circRNAs | Number of circRNAs | Minimum size, bp | Maximum size, bp | |
| NC_031987.2 | 26789386 | 26792503 | XM_019352997.1 | XM_019352997.1 PREDICTED: Oreochromis niloticus nebulin (neb), transcript variant X7, mRNA | 1 | 3148 | 3148 | |
| NC_031986.2 | 11263255 | 11266860 | XM_019351651.1 | XM_019351651.1 PREDICTED: Oreochromis niloticus muscleblind-like protein 2a (LOC100695201), transcript variant X5, mRNA | 1 | 3636 | 3636 | |
| NC_031984.2 | 4519550 | 4524223 | XM_019349722.1 | XM_019349722.1 PREDICTED: Oreochromis niloticus solute carrier family 6 member 8 (slc6a8), transcript variant X3, mRNA | 1 | 4702 | 4702 | |
| NC_031983.2 | 22346989 | 22348856 | XM_019348631.1 | XM_019348631.1 PREDICTED: Oreochromis niloticus solute carrier family 8 member A3 (slc8a3), transcript variant X3, mRNA | 1 | 1898 | 1898 | |
| NC_031982.2 | 28729732 | 28739609 | XM_019348080.1 | XM_019348080.1 PREDICTED: Oreochromis niloticus partitioning defective 3 homolog (LOC100698856), transcript variant X12, mRNA | 1 | 9908 | 9908 | |
| NC_031982.2 | 27199427 | 27203702 | XM_019348045.1 | XM_019348045.1 PREDICTED: Oreochromis niloticus supervillin (LOC100703985), transcript variant X11, mRNA | 1 | 4306 | 4306 | |
| NC_031982.2 | 15053320 | 15093159 | AB505452.1 | AB505452.1 Rana rugosa got1 gene for glutamate oxaloacetate transaminase, partial cds | 1 | 39870 | 39870 | |
| NC_031982.2 | 14453483 | 14455763 | XM_019347703.1 | XM_019347703.1 PREDICTED: Oreochromis niloticus muscleblind-like protein 1 (LOC100712433), transcript variant X21, mRNA | 1 | 2311 | 2311 | |
| NC_031982.2 | 13444988 | 13469921 | XM_005476190.3 | XM_005476190.3 PREDICTED: Oreochromis niloticus myomegalin (LOC100703940), transcript variant X7, mRNA | 1 | 24964 | 24964 | |
| NC_031980.2 | 10790463 | 10804951 | XR_001224849.2 | XR_001224849.2 PREDICTED: Oreochromis niloticus SAM and SH3 domain containing 1 (sash1), transcript variant X2, misc_RNA | 1 | 14519 | 14519 | |
| NC_031979.2 | 36408021 | 36408711 | XM_012915678.2 | XM_012915678.2 PREDICTED: Maylandia zebra actinin, alpha 4 (actn4), transcript variant X7, mRNA | 1 | 721 | 721 | |
| NC_031979.2 | 24606047 | 24609702 | XM_005459298.3 | XM_005459298.3 PREDICTED: Oreochromis niloticus membrane metalloendopeptidase (mme), transcript variant X4, mRNA | 1 | 3686 | 3686 | |
| NC_031978.2 | 30702661 | 30704116 | XM_005461429.3 | XM_005461429.3 PREDICTED: Oreochromis niloticus piezo type mechanosensitive ion channel component 1 (piezo1), mRNA | 1 | 1486 | 1486 | |
| NC_031978.2 | 102602 | 105225 | AB270897.1 | AB270897.1 Oreochromis niloticus MHC class IA antigen UBA1, UBA2, UAA1 genes, partial cds, UAA3 and UAA2 pseudogenes, UAA4, UAA5 and UAA6 pseudogene fragments | 1 | 2650 | 2650 | |
| NC_031976.2 | 381140 | 383383 | XM_019354539.1 | XM_019354539.1 PREDICTED: Oreochromis niloticus activating signal cointegrator 1 complex subunit 3 (ascc3), transcript variant X5, mRNA | 1 | 2270 | 2270 | |
| NC_031973.2 | 1854460 | 1854640 | XM_006793296.1 | NOT ASSIGNED | 1 | 209 | 209 | |
| NC_031972.2 | 9033630 | 9035959 | XM_019360763.1 | XM_019360763.1 PREDICTED: Oreochromis niloticus myocyte-specific enhancer factor 2C (LOC100706088), transcript variant X4, mRNA | 1 | 2358 | 2358 | |
| NC_031971.2 | 27059186 | 27061091 | XM_006787490.1 | XM_006787490.1 PREDICTED: Neolamprologus brichardi ubinuclein-2-like (LOC102799674), mRNA | 1 | 1936 | 1936 | |
| NC_031967.2 | 7417222 | 7418306 | XM_005455235.3 | XM_005455235.3 PREDICTED: Oreochromis niloticus fibroblast growth factor 11 (fgf11), transcript variant X2, mRNA | 1 | 1113 | 1113 | |
|  | 23491153 | 23491752 |  | NOT ASSIGNED | 1 | 630 | 630 | |
| Gene ID | Gene coordinates start | Gene coordinates end | Gene of origin for circRNAs ID | Gene of origin for circRNAs | Number of circRNAs | Minimum size, bp | Maximum size, bp | |
| NC_031987.2 | 26790674 | 26791360 | XM_013914149.1 | XM_013914149.1 PREDICTED: Pundamilia nyererei nebulin (neb), mRNA | 1 | 717 | 717 | |
| NC_031987.2 | 21667160 | 21673355 | AB270897.1 | AB270897.1 Oreochromis niloticus MHC class IA antigen UBA1, UBA2, UAA1 genes, partial cds, UAA3 and UAA2 pseudogenes, UAA4, UAA5 and UAA6 pseudogene fragments | 1 | 6226 | 6226 | |
| NC_031986.2 | 45119249 | 45119970 | XM_013268343.2 | XM_013268343.2 PREDICTED: Oreochromis niloticus nexilin F-actin binding protein (nexn), transcript variant X3, mRNA | 1 | 752 | 752 | |
| NC_031986.2 | 35080413 | 35089898 | XM_019351928.1 | XM_019351928.1 PREDICTED: Oreochromis niloticus semaphorin 6B (sema6b), transcript variant X5, mRNA | 1 | 9516 | 9516 | |
| NC_031986.2 | 2525332 | 2526568 | XM_013277247.2 | XM_013277247.2 PREDICTED: Oreochromis niloticus dystrophin (LOC100700501), transcript variant X4, mRNA | 1 | 1265 | 1265 | |
| NC_031986.2 | 11742381 | 11743817 | XM_013268880.2 | XM_013268880.2 PREDICTED: Oreochromis niloticus 5'-AMP-activated protein kinase subunit gamma-1 (LOC100697765), transcript variant X5, mRNA | 1 | 1467 | 1467 | |
| NC_031985.2 | 21224875 | 21231611 | XM_003444072.4 | XM_003444072.4 PREDICTED: Oreochromis niloticus CAP, adenylate cyclase-associated protein, 2 (yeast) (cap2), mRNA | 1 | 6767 | 6767 | |
| NC_031984.2 | 22067641 | 22068248 | XM_005478204.3 | XM_005478204.3 PREDICTED: Oreochromis niloticus calsyntenin 1 (clstn1), transcript variant X3, mRNA | 1 | 638 | 638 | |
| NC_031984.2 | 15224199 | 15225087 | XM_014336629.1 | XM_014336629.1 PREDICTED: Haplochromis burtoni glutamate receptor-interacting protein 2-like (LOC102303775), transcript variant X6, mRNA | 1 | 919 | 919 | |
| NC_031983.2 | 4961483 | 4975778 | XM_019348923.1 | XM_019348923.1 PREDICTED: Oreochromis niloticus A-kinase anchoring protein 6 (akap6), transcript variant X1, mRNA | 1 | 14324 | 14324 | |
| NC_031982.2 | 34115451 | 34142233 | XM_005464201.3 | XM_005464201.3 PREDICTED: Oreochromis niloticus ras GTPase-activating protein nGAP (LOC100701601), mRNA | 1 | 26813 | 26813 | |
| NC_031982.2 | 27199427 | 27203702 | XM_019348045.1 | XM_019348045.1 PREDICTED: Oreochromis niloticus supervillin (LOC100703985), transcript variant X11, mRNA | 1 | 4306 | 4306 | |
| NC_031982.2 | 15066734 | 15106042 | XM_005476264.3 | XM_005476264.3 PREDICTED: Oreochromis niloticus glypican-5 (LOC102077503), transcript variant X2, mRNA | 1 | 39339 | 39339 | |
| NC_031981.2 | 12545456 | 12549945 | XM_019347073.1 | XM_019347073.1 PREDICTED: Oreochromis niloticus voltage-dependent calcium channel subunit alpha-2/delta-1 (LOC100705144), transcript variant X5, mRNA | 1 | 4520 | 4520 | |
| NC_031981.2 | 10272055 | 10281829 | XR_001224370.2 | XR_001224370.2 PREDICTED: Oreochromis niloticus ELKS/Rab6-interacting/CAST family member 1 (LOC100690550), transcript variant X10, misc_RNA | 1 | 9805 | 9805 | |
| NC_031980.2 | 896114 | 897346 | XR_002058847.1 | XR_002058847.1 PREDICTED: Oreochromis niloticus CDC42 binding protein kinase alpha (cdc42bpa), transcript variant X2, misc_RNA | 1 | 1259 | 1259 | |
| NC_031980.2 | 33724195 | 33729310 | XM_019345876.1 | XM_019345876.1 PREDICTED: Oreochromis niloticus receptor-type tyrosine-protein phosphatase S (LOC100702908), transcript variant X15, mRNA | 1 | 5146 | 5146 | |
| NC_031979.2 | 35013862 | 35016025 | XM_013913810.1 | XM_013913810.1 PREDICTED: Pundamilia nyererei hypoxia-inducible factor 1-alpha-like (LOC102195817), mRNA | 1 | 2194 | 2194 | |
| NC_031979.2 | 24606047 | 24609702 | XM_005459298.3 | XM_005459298.3 PREDICTED: Oreochromis niloticus membrane metalloendopeptidase (mme), transcript variant X4, mRNA | 1 | 3686 | 3686 | |
| NC_031978.2 | 30702661 | 30704116 | XM_005461429.3 | XM_005461429.3 PREDICTED: Oreochromis niloticus piezo type mechanosensitive ion channel component 1 (piezo1), mRNA | 1 | 1486 | 1486 | |
| NC_031978.2 | 25214297 | 25216302 | XM_019366502.1 | XM_019366502.1 PREDICTED: Oreochromis niloticus leucine-rich repeat flightless-interacting protein 2 (LOC100700265), transcript variant X7, mRNA | 1 | 2036 | 2036 | |
| NC_031977.2 | 20038517 | 20040903 | XR_266173.3 | XR_266173.3 PREDICTED: Oreochromis niloticus sperm tail PG-rich repeat containing 2 (stpg2), transcript variant X3, misc_RNA | 1 | 2417 | 2417 | |
| NC_031977.2 | 19508827 | 19509068 | XM_019365292.1 | XM_019365292.1 PREDICTED: Oreochromis niloticus ubiquitin-associated protein 2 (LOC100696291), transcript variant X4, mRNA | 1 | 272 | 272 | |
| NC_031977.2 | 15722348 | 15722913 | XM_019365621.1 | XM_019365621.1 PREDICTED: Oreochromis niloticus rho-related BTB domain-containing protein 2 (LOC100707935), transcript variant X5, mRNA | 1 | 596 | 596 | |
| NC_031976.2 | 4617931 | 4618143 | XR_002063780.1 | XR_002063780.1 PREDICTED: Oreochromis niloticus SNF related kinase (snrk), transcript variant X2, misc_RNA | 1 | 241 | 241 | |
| NC_031976.2 | 22399782 | 22400576 | XM_005455378.3 | XM_005455378.3 PREDICTED: Oreochromis niloticus myocardin-like (LOC102078953), mRNA | 1 | 825 | 825 | |
| NC_031975.2 | 18038285 | 18039720 | XM_005452672.3 | XM_005452672.3 PREDICTED: Oreochromis niloticus protein NipSnap homolog 2 (LOC100704964), mRNA | 1 | 1466 | 1466 | |
| NC_031973.2 | 4626128 | 4627595 | XM_019362328.1 | XM_019362328.1 PREDICTED: Oreochromis niloticus KAT8 regulatory NSL complex subunit 1 (kansl1), transcript variant X5, mRNA | 1 | 1496 | 1496 | |
| NC_031973.2 | 28425174 | 28426156 | XR_269440.3 | XR_269440.3 PREDICTED: Oreochromis niloticus RFNG O-fucosylpeptide 3-beta-N-acetylglucosaminyltransferase (rfng), transcript variant X2, misc_RNA | 1 | 1013 | 1013 | |
| NC_031973.2 | 1854460 | 1854640 | XM_006793296.1 | NOT ASSIGNED | 1 | 209 | 209 | |
| NC_031972.2 | 9033630 | 9035959 | XM_019360763.1 | XM_019360763.1 PREDICTED: Oreochromis niloticus myocyte-specific enhancer factor 2C (LOC100706088), transcript variant X4, mRNA | 1 | 2358 | 2358 | |
| NC_031972.2 | 61245143 | 61245486 | XM_019361891.1 | XM_019361891.1 PREDICTED: Oreochromis niloticus microtubule associated monooxygenase, calponin and LIM domain containing 3 (mical3), transcript variant X3, mRNA | 1 | 374 | 374 | |
| NC_031972.2 | 53518480 | 53519879 | XM_019361602.1 | XM_019361602.1 PREDICTED: Oreochromis niloticus chromodomain helicase DNA binding protein 2 (chd2), transcript variant X5, mRNA | 1 | 1430 | 1430 | |
| NC_031972.2 | 48409310 | 48413963 | XM_014335746.1 | XM_014335746.1 PREDICTED: Haplochromis burtoni CUGBP Elav-like family member 2 (LOC102310863), mRNA | 1 | 4684 | 4684 | |
| NC_031972.2 | 3583995 | 3584602 | XM_005449241.2 | XM_005449241.2 PREDICTED: Oreochromis niloticus troponin T3, fast skeletal type (tnnt3), transcript variant X6, mRNA | 1 | 636 | 636 | |
| NC_031971.2 | 42021773 | 42028544 | AB270897.1 | AB270897.1 Oreochromis niloticus MHC class IA antigen UBA1, UBA2, UAA1 genes, partial cds, UAA3 and UAA2 pseudogenes, UAA4, UAA5 and UAA6 pseudogene fragments | 1 | 6802 | 6802 | |
| NC_031971.2 | 4075465 | 4076159 | XM_005461334.3 | XM_005461334.3 PREDICTED: Oreochromis niloticus endoplasmic reticulum oxidoreductase 1 beta (ero1b), transcript variant X2, mRNA | 1 | 723 | 723 | |
| NC_031971.2 | 27059186 | 27061091 | XM_006787490.1 | XM_006787490.1 PREDICTED: Neolamprologus brichardi ubinuclein-2-like (LOC102799674), mRNA | 1 | 1936 | 1936 | |
| NC_031970.2 | 32666106 | 32668191 | XM_005450143.3 | XM_005450143.3 PREDICTED: Oreochromis niloticus PDZ domain containing ring finger 3 (pdzrn3), transcript variant X2, mRNA | 1 | 2116 | 2116 | |
| NC_031969.2 | 23491752 | 23492386 | XM_019357829.1 | XM_019357829.1 PREDICTED: Oreochromis niloticus myosin heavy chain, fast skeletal muscle (LOC100712344), mRNA | 1 | 665 | 665 | |
| NC_031967.2 | 32532813 | 32533411 | XM_019353059.1 | XM_019353059.1 PREDICTED: Oreochromis niloticus phosphorylase b kinase regulatory subunit alpha, skeletal muscle isoform (LOC100707493), transcript variant X12, mRNA | 1 | 629 | 629 | |
| NC_031967.2 | 3188565 | 3190679 | XM_019349016.1 | XM_019349016.1 PREDICTED: Oreochromis niloticus echinoderm microtubule associated protein like 3 (eml3), transcript variant X2, mRNA | 1 | 2143 | 2143 | |
| NC_031966.2 | 29940845 | 29941924 | XM_005467991.3 | XM_005467991.3 PREDICTED: Oreochromis niloticus SH3 domain and tetratricopeptide repeats 2 (sh3tc2), transcript variant X2, mRNA | 1 | 1110 | 1110 | |
| NC_031965.2 | 8872238 | 8885885 | XM_019349184.1 | XM_019349184.1 PREDICTED: Oreochromis niloticus calpain-1 catalytic subunit (LOC106098300), transcript variant X2, mRNA | 1 | 13676 | 13676 | |
| Gene ID | Gene coordinates start | Gene coordinates end | Gene of origin for circRNAs ID | Gene of origin for circRNAs | Number of circRNAs | Minimum size, bp | Maximum size, bp | |
| NC_031984.2 | 35582665 | 35589644 | AH013711.2 | AH013711.2 Oreochromis niloticus KLR3 (KLR3) gene, exons 2 through 7; KLR2 pseudogene, partial sequence; KLR1 gene, complete sequence; KLR4 (KLR4) gene, exons 2 through 7; KLR5 (KLR5) gene, exons 1 through 7; KLR6 and KLR7 pseudogenes, complete sequence; KLR9 (KLR10) pseudogene, exons 4 through 7; KLR8 pseudogene, partial sequence; KLR9 (KLR9) gene, exons 1 through 7; C-type lectin (CLECT2)-like protein gene, complete sequence; C-type lectin natural killer cell receptor-like protein gene, exons 1 and 2; and transposon TX1-like ORF2 pseudogene, partial sequence | 1 | 7010 | 7010 | |
| NC_031984.2 | 29112026 | 29113572 | XR_002057744.1 | XR_002057744.1 PREDICTED: Oreochromis niloticus vitamin D3 receptor A (LOC100696631), transcript variant X2, misc_RNA | 1 | 1577 | 1577 | |
| NC_031984.2 | 15224199 | 15225087 | XM_014336629.1 | XM_014336629.1 PREDICTED: Haplochromis burtoni glutamate receptor-interacting protein 2-like (LOC102303775), transcript variant X6, mRNA | 1 | 919 | 919 | |
| NC_031983.2 | 30813628 | 30814989 | XM_005942849.2 | XM_005942849.2 PREDICTED: Haplochromis burtoni islet cell autoantigen 1, 69kDa (ica1), mRNA | 1 | 1392 | 1392 | |
| NC_031981.2 | 12545456 | 12549945 | XM_019347073.1 | XM_019347073.1 PREDICTED: Oreochromis niloticus voltage-dependent calcium channel subunit alpha-2/delta-1 (LOC100705144), transcript variant X5, mRNA | 1 | 4520 | 4520 | |
| NC_031981.2 | 10272056 | 10281829 | XR_001224370.2 | XR_001224370.2 PREDICTED: Oreochromis niloticus ELKS/Rab6-interacting/CAST family member 1 (LOC100690550), transcript variant X10, misc_RNA | 1 | 9804 | 9804 | |
| NC_031980.2 | 896114 | 897054 | XR_002058847.1 | XR_002058847.1 PREDICTED: Oreochromis niloticus CDC42 binding protein kinase alpha (cdc42bpa), transcript variant X2, misc_RNA | 1 | 967 | 967 | |
| NC_031980.2 | 32787027 | 32789391 | XM_019346232.1 | XM_019346232.1 PREDICTED: Oreochromis niloticus transmembrane protein 38A (tmem38a), transcript variant X2, mRNA | 1 | 2395 | 2395 | |
| NC_031980.2 | 10790463 | 10804951 | XR_001224849.2 | XR_001224849.2 PREDICTED: Oreochromis niloticus SAM and SH3 domain containing 1 (sash1), transcript variant X2, misc_RNA | 1 | 14519 | 14519 | |
| NC_031979.2 | 5190613 | 5198088 | XM_019353524.1 | XM_019353524.1 PREDICTED: Oreochromis niloticus toll like receptor 5 (tlr5), transcript variant X2, mRNA | 1 | 7504 | 7504 | |
| NC_031979.2 | 35013862 | 35016025 | XM_013913810.1 | XM_013913810.1 PREDICTED: Pundamilia nyererei hypoxia-inducible factor 1-alpha-like (LOC102195817), mRNA | 1 | 2194 | 2194 | |
| NC_031979.2 | 24606047 | 24609702 | XM_005459298.3 | XM_005459298.3 PREDICTED: Oreochromis niloticus membrane metalloendopeptidase (mme), transcript variant X4, mRNA | 1 | 3686 | 3686 | |
| NC_031979.2 | 23188464 | 23189463 | XM_005459369.3 | XM_005459369.3 PREDICTED: Oreochromis niloticus cut like homeobox 1 (cux1), transcript variant X2, mRNA | 1 | 1030 | 1030 | |
| NC_031978.2 | 30702661 | 30704116 | XM_005461429.3 | XM_005461429.3 PREDICTED: Oreochromis niloticus piezo type mechanosensitive ion channel component 1 (piezo1), mRNA | 1 | 1486 | 1486 | |
| NC_031978.2 | 25214297 | 25216302 | XM_019366502.1 | XM_019366502.1 PREDICTED: Oreochromis niloticus leucine-rich repeat flightless-interacting protein 2 (LOC100700265), transcript variant X7, mRNA | 1 | 2036 | 2036 | |
| NC_031976.2 | 95036 | 95722 | XR_002059667.1 | XR_002059667.1 PREDICTED: Oreochromis niloticus centrosomal protein 162 (cep162), transcript variant X11, misc_RNA | 1 | 711 | 711 | |
| NC_031976.2 | 4608763 | 4618143 | XM_019358987.1 | XM_019358987.1 PREDICTED: Oreochromis niloticus RNA-directed DNA polymerase from mobile element jockey-like (LOC109202218), partial mRNA | 1 | 9409 | 9409 | |
| NC_031972.2 | 9033630 | 9035959 | XM_019360763.1 | XM_019360763.1 PREDICTED: Oreochromis niloticus myocyte-specific enhancer factor 2C (LOC100706088), transcript variant X4, mRNA | 1 | 2358 | 2358 | |
| NC_031972.2 | 61245143 | 61245486 | XM_019361891.1 | XM_019361891.1 PREDICTED: Oreochromis niloticus microtubule associated monooxygenase, calponin and LIM domain containing 3 (mical3), transcript variant X3, mRNA | 1 | 374 | 374 | |
| NC_031972.2 | 48409310 | 48413963 | XM_014335746.1 | XM_014335746.1 PREDICTED: Haplochromis burtoni CUGBP Elav-like family member 2 (LOC102310863), mRNA | 1 | 4684 | 4684 | |
| NC_031972.2 | 3583995 | 3584602 | XM_005449241.2 | XM_005449241.2 PREDICTED: Oreochromis niloticus troponin T3, fast skeletal type (tnnt3), transcript variant X6, mRNA | 1 | 636 | 636 | |
| NC_031971.2 | 4075465 | 4076159 | XM_005461334.3 | XM_005461334.3 PREDICTED: Oreochromis niloticus endoplasmic reticulum oxidoreductase 1 beta (ero1b), transcript variant X2, mRNA | 1 | 723 | 723 | |
| NC_031971.2 | 27059186 | 27061091 | XM_006787490.1 | XM_006787490.1 PREDICTED: Neolamprologus brichardi ubinuclein-2-like (LOC102799674), mRNA | 1 | 1936 | 1936 | |
| NC_031970.2 | 34653249 | 34658229 | AB270897.1 | AB270897.1 Oreochromis niloticus MHC class IA antigen UBA1, UBA2, UAA1 genes, partial cds, UAA3 and UAA2 pseudogenes, UAA4, UAA5 and UAA6 pseudogene fragments | 1 | 5011 | 5011 | |
| NC_031970.2 | 30421868 | 30422532 | XM_003442657.4 | XM_003442657.4 PREDICTED: Oreochromis niloticus RNA-binding protein 38 (LOC100702167), mRNA | 1 | 695 | 695 | |
| NC_031969.2 | 35390737 | 35394011 | XM_019357502.1 | XM_019357502.1 PREDICTED: Oreochromis niloticus cell division cycle 27 (cdc27), transcript variant X2, mRNA | 1 | 3305 | 3305 | |
| NC_031966.2 | 29940845 | 29941924 | XM_005467991.3 | XM_005467991.3 PREDICTED: Oreochromis niloticus SH3 domain and tetratricopeptide repeats 2 (sh3tc2), transcript variant X2, mRNA | 1 | 1110 | 1110 | |
| Gene ID | Gene coordinates start | Gene coordinates end | Gene of origin for circRNAs ID | Gene of origin for circRNAs | Number of circRNAs | Minimum size, bp | Maximum size, bp | |
| NC_031987.2 | 35831744 | 35836495 | XM_019353050.1 | XM_019353050.1 PREDICTED: Oreochromis niloticus ras-associated and pleckstrin homology domains-containing protein 1 (LOC100710818), mRNA | 1 | 4782 | 4782 | |
| NC_031987.2 | 26790951 | 26792062 | XM_019352997.1 | XM_019352997.1 PREDICTED: Oreochromis niloticus nebulin (neb), transcript variant X7, mRNA | 1 | 1142 | 1142 | |
| NC_031986.2 | 45119249 | 45119970 | XM_013268343.2 | XM_013268343.2 PREDICTED: Oreochromis niloticus nexilin F-actin binding protein (nexn), transcript variant X3, mRNA | 1 | 752 | 752 | |
| NC_031984.2 | 31652472 | 31653850 | XM_006804056.1 | XM_006804056.1 PREDICTED: Neolamprologus brichardi troponin C, skeletal muscle-like (LOC102782874), mRNA | 1 | 1409 | 1409 | |
| NC_031984.2 | 22067641 | 22068248 | XM_005478204.3 | XM_005478204.3 PREDICTED: Oreochromis niloticus calsyntenin 1 (clstn1), transcript variant X3, mRNA | 1 | 638 | 638 | |
| NC_031984.2 | 15224199 | 15225087 | XM_014336629.1 | XM_014336629.1 PREDICTED: Haplochromis burtoni glutamate receptor-interacting protein 2-like (LOC102303775), transcript variant X6, mRNA | 1 | 919 | 919 | |
| NC_031983.2 | 9184286 | 9188456 | XM_005453302.2 | XM_005453302.2 PREDICTED: Oreochromis niloticus cysteine rich transmembrane BMP regulator 1 (crim1), transcript variant X3, mRNA | 1 | 4199 | 4199 | |
| NC_031982.2 | 28721832 | 28740958 | XM_019348080.1 | XM_019348080.1 PREDICTED: Oreochromis niloticus partitioning defective 3 homolog (LOC100698856), transcript variant X12, mRNA | 1 | 19157 | 19157 | |
| NC_031982.2 | 27199427 | 27203702 | XM_019348045.1 | XM_019348045.1 PREDICTED: Oreochromis niloticus supervillin (LOC100703985), transcript variant X11, mRNA | 1 | 4306 | 4306 | |
| NC_031982.2 | 23105201 | 23105671 | XM_003448490.3 | XM_003448490.3 PREDICTED: Oreochromis niloticus complexin-4 (LOC100705335), transcript variant X1, mRNA | 1 | 501 | 501 | |
| NC_031982.2 | 22933984 | 22934532 | XM_005476595.3 | XM_005476595.3 PREDICTED: Oreochromis niloticus insulin receptor (LOC100696191), mRNA | 1 | 579 | 579 | |
| NC_031982.2 | 15091152 | 15093159 | XM_005476264.3 | XM_005476264.3 PREDICTED: Oreochromis niloticus glypican-5 (LOC102077503), transcript variant X2, mRNA | 1 | 2038 | 2038 | |
| NC_031981.2 | 12545456 | 12549945 | XM_019347073.1 | XM_019347073.1 PREDICTED: Oreochromis niloticus voltage-dependent calcium channel subunit alpha-2/delta-1 (LOC100705144), transcript variant X5, mRNA | 1 | 4520 | 4520 | |
| NC_031981.2 | 10272055 | 10281829 | XR_001224370.2 | XR_001224370.2 PREDICTED: Oreochromis niloticus ELKS/Rab6-interacting/CAST family member 1 (LOC100690550), transcript variant X10, misc_RNA | 1 | 9805 | 9805 | |
| NC_031980.2 | 896114 | 897054 | XR_002058847.1 | XR_002058847.1 PREDICTED: Oreochromis niloticus CDC42 binding protein kinase alpha (cdc42bpa), transcript variant X2, misc_RNA | 1 | 967 | 967 | |
| NC_031980.2 | 8216973 | 8219849 | XR_002056582.1 | XR_002056582.1 PREDICTED: Oreochromis niloticus EYA transcriptional coactivator and phosphatase 4 (eya4), transcript variant X3, misc_RNA | 1 | 2905 | 2905 | |
| NC_031980.2 | 4516125 | 4523685 | XM_019346164.1 | XM_019346164.1 PREDICTED: Oreochromis niloticus MAX gene-associated protein (LOC100697049), transcript variant X4, mRNA | 1 | 7589 | 7589 | |
| NC_031979.2 | 35013862 | 35016025 | XM_013913810.1 | XM_013913810.1 PREDICTED: Pundamilia nyererei hypoxia-inducible factor 1-alpha-like (LOC102195817), mRNA | 1 | 2194 | 2194 | |
| NC_031979.2 | 24606047 | 24609702 | XM_005459298.3 | XM_005459298.3 PREDICTED: Oreochromis niloticus membrane metalloendopeptidase (mme), transcript variant X4, mRNA | 1 | 3686 | 3686 | |
| NC_031978.2 | 4966624 | 4969901 | XM_019366814.1 | XM_019366814.1 PREDICTED: Oreochromis niloticus attractin-like protein 1 (LOC100692310), transcript variant X3, mRNA | 1 | 3306 | 3306 | |
| NC_031978.2 | 30759710 | 30763908 | XM_005461429.3 | XM_005461429.3 PREDICTED: Oreochromis niloticus piezo type mechanosensitive ion channel component 1 (piezo1), mRNA | 1 | 4229 | 4229 | |
| NC_031978.2 | 30702661 | 30704116 | XM_005461429.3 | XM_005461429.3 PREDICTED: Oreochromis niloticus piezo type mechanosensitive ion channel component 1 (piezo1), mRNA | 1 | 1486 | 1486 | |
| NC_031978.2 | 25214297 | 25216302 | XM_019366502.1 | XM_019366502.1 PREDICTED: Oreochromis niloticus leucine-rich repeat flightless-interacting protein 2 (LOC100700265), transcript variant X7, mRNA | 1 | 2036 | 2036 | |
| NC_031978.2 | 102602 | 105225 | AB270897.1 | AB270897.1 Oreochromis niloticus MHC class IA antigen UBA1, UBA2, UAA1 genes, partial cds, UAA3 and UAA2 pseudogenes, UAA4, UAA5 and UAA6 pseudogene fragments | 1 | 2650 | 2650 | |
| NC_031977.2 | 20038517 | 20040903 | XR_266173.3 | XR_266173.3 PREDICTED: Oreochromis niloticus sperm tail PG-rich repeat containing 2 (stpg2), transcript variant X3, misc_RNA | 1 | 2417 | 2417 | |
| NC_031977.2 | 19508827 | 19509068 | XM_019365292.1 | XM_019365292.1 PREDICTED: Oreochromis niloticus ubiquitin-associated protein 2 (LOC100696291), transcript variant X4, mRNA | 1 | 272 | 272 | |
| NC_031976.2 | 95036 | 95722 | XR_002059667.1 | XR_002059667.1 PREDICTED: Oreochromis niloticus centrosomal protein 162 (cep162), transcript variant X11, misc_RNA | 1 | 711 | 711 | |
| NC_031976.2 | 4608763 | 4618143 | XM_019358987.1 | XM_019358987.1 PREDICTED: Oreochromis niloticus RNA-directed DNA polymerase from mobile element jockey-like (LOC109202218), partial mRNA | 1 | 9409 | 9409 | |
| NC_031976.2 | 36016595 | 36018688 | XM_013275037.2 | XM_013275037.2 PREDICTED: Oreochromis niloticus lamin A/C (lmna), transcript variant X2, mRNA | 1 | 2124 | 2124 | |
| NC_031976.2 | 283826 | 284149 | XM_019354539.1 | XM_019354539.1 PREDICTED: Oreochromis niloticus activating signal cointegrator 1 complex subunit 3 (ascc3), transcript variant X5, mRNA | 1 | 350 | 350 | |
| NC_031975.2 | 7731058 | 7732347 | XM_003450463.4 | XM_003450463.4 PREDICTED: Oreochromis niloticus seizure protein 6 homolog (LOC100703088), mRNA | 1 | 1318 | 1318 | |
| NC_031974.2 | 29672184 | 29675582 | XM_019362958.1 | XM_019362958.1 PREDICTED: Oreochromis niloticus supervillin (svil), transcript variant X17, mRNA | 1 | 3429 | 3429 | |
| NC_031973.2 | 9903733 | 9905025 | XM_019362446.1 | XM_019362446.1 PREDICTED: Oreochromis niloticus eukaryotic elongation factor 2 kinase (eef2k), transcript variant X7, mRNA | 1 | 1321 | 1321 | |
| NC_031973.2 | 4626128 | 4627595 | XM_019362328.1 | XM_019362328.1 PREDICTED: Oreochromis niloticus KAT8 regulatory NSL complex subunit 1 (kansl1), transcript variant X5, mRNA | 1 | 1496 | 1496 | |
| NC_031972.2 | 9033630 | 9035959 | XM_019360763.1 | XM_019360763.1 PREDICTED: Oreochromis niloticus myocyte-specific enhancer factor 2C (LOC100706088), transcript variant X4, mRNA | 1 | 2358 | 2358 | |
| NC_031972.2 | 63125120 | 63127881 | XM_005471149.3 | XM_005471149.3 PREDICTED: Oreochromis niloticus G1/S-specific cyclin-D2 (LOC100698323), transcript variant X2, mRNA | 1 | 2792 | 2792 | |
| NC_031972.2 | 61270809 | 61273681 | XM_019361915.1 | XM_019361915.1 PREDICTED: Oreochromis niloticus microtubule associated monooxygenase, calponin and LIM domain containing 3 (mical3), transcript variant X28, mRNA | 1 | 2903 | 2903 | |
| NC_031972.2 | 61245143 | 61245486 | XM_019361891.1 | XM_019361891.1 PREDICTED: Oreochromis niloticus microtubule associated monooxygenase, calponin and LIM domain containing 3 (mical3), transcript variant X3, mRNA | 1 | 374 | 374 | |
| NC_031972.2 | 48409310 | 48413963 | XM_014335746.1 | XM_014335746.1 PREDICTED: Haplochromis burtoni CUGBP Elav-like family member 2 (LOC102310863), mRNA | 1 | 4684 | 4684 | |
| NC_031972.2 | 41189270 | 41189586 | XM_019357934.1 | XM_019357934.1 PREDICTED: Oreochromis niloticus suppression of tumorigenicity 7 (st7), transcript variant X1, mRNA | 1 | 347 | 347 | |
| NC_031972.2 | 39782274 | 39784019 | XM_019361198.1 | XM_019361198.1 PREDICTED: Oreochromis niloticus anoctamin-1 (LOC100704804), transcript variant X2, mRNA | 1 | 1776 | 1776 | |
| NC_031972.2 | 3583995 | 3584602 | XM_005449241.2 | XM_005449241.2 PREDICTED: Oreochromis niloticus troponin T3, fast skeletal type (tnnt3), transcript variant X6, mRNA | 1 | 636 | 636 | |
| NC_031971.2 | 42021773 | 42028544 | AB270897.1 | AB270897.1 Oreochromis niloticus MHC class IA antigen UBA1, UBA2, UAA1 genes, partial cds, UAA3 and UAA2 pseudogenes, UAA4, UAA5 and UAA6 pseudogene fragments | 1 | 6802 | 6802 | |
| NC_031971.2 | 4075465 | 4076159 | XM_005461334.3 | XM_005461334.3 PREDICTED: Oreochromis niloticus endoplasmic reticulum oxidoreductase 1 beta (ero1b), transcript variant X2, mRNA | 1 | 723 | 723 | |
| NC_031971.2 | 27059186 | 27061091 | XM_006787490.1 | XM_006787490.1 PREDICTED: Neolamprologus brichardi ubinuclein-2-like (LOC102799674), mRNA | 1 | 1936 | 1936 | |
| NC_031971.2 | 24932987 | 24933518 | XM_013272974.2 | XM_013272974.2 PREDICTED: Oreochromis niloticus nuclear factor 1 X-type (LOC100690838), transcript variant X14, mRNA | 1 | 562 | 562 | |
| NC_031967.2 | 32532813 | 32533411 | XM_019353059.1 | XM_019353059.1 PREDICTED: Oreochromis niloticus phosphorylase b kinase regulatory subunit alpha, skeletal muscle isoform (LOC100707493), transcript variant X12, mRNA | 1 | 629 | 629 | |
| NC_031967.2 | 3188565 | 3190679 | XM_019349016.1 | XM_019349016.1 PREDICTED: Oreochromis niloticus echinoderm microtubule associated protein like 3 (eml3), transcript variant X2, mRNA | 1 | 2143 | 2143 | |
| NC_031967.2 | 23990398 | 23992907 | XM_019356887.1 | XM_019356887.1 PREDICTED: Oreochromis niloticus phosphofurin acidic cluster sorting protein 1 (pacs1), transcript variant X2, mRNA | 1 | 2540 | 2540 | |
| NC_031966.2 | 29940845 | 29941924 | XM_005467991.3 | XM_005467991.3 PREDICTED: Oreochromis niloticus SH3 domain and tetratricopeptide repeats 2 (sh3tc2), transcript variant X2, mRNA | 1 | 1110 | 1110 | |
| NC_031966.2 | 25348411 | 25349729 | XM_012924841.2 | XM_012924841.2 PREDICTED: Maylandia zebra myozenin-2-like (LOC101481626), mRNA | 1 | 1349 | 1349 | |
| NC_031966.2 | 21354417 | 21356032 | XM_006781341.1 | XM_006781341.1 PREDICTED: Neolamprologus brichardi probable E3 ubiquitin-protein ligase MID2-like (LOC102785246), transcript variant X5, mRNA | 1 | 1646 | 1646 | |
| NC_031965.2 | 8872238 | 8885885 | XM_019349184.1 | XM_019349184.1 PREDICTED: Oreochromis niloticus calpain-1 catalytic subunit (LOC106098300), transcript variant X2, mRNA | 1 | 13676 | 13676 | |
| Gene ID | Gene coordinates start | Gene coordinates end | Gene of origin for circRNAs ID | Gene of origin for circRNAs | Number of circRNAs | Minimum size, bp | Maximum size, bp | |
| NC_031987.2 | 26789386 | 26791055 | XM_019352997.1 | XM_019352997.1 PREDICTED: Oreochromis niloticus nebulin (neb), transcript variant X7, mRNA | 1 | 1700 | 1700 | |
| NC_031987.2 | 14996343 | 15015105 | XM_019353022.1 | XM_019353022.1 PREDICTED: Oreochromis niloticus plakophilin 4 (pkp4), transcript variant X4, mRNA | 1 | 18793 | 18793 | |
| NC_031986.2 | 45119249 | 45119970 | XM_013268343.2 | XM_013268343.2 PREDICTED: Oreochromis niloticus nexilin F-actin binding protein (nexn), transcript variant X3, mRNA | 1 | 752 | 752 | |
| NC_031986.2 | 3950837 | 3954216 | XM_019351894.1 | XM_019351894.1 PREDICTED: Oreochromis niloticus dystrophin (LOC100694991), mRNA | 1 | 3408 | 3408 | |
| NC_031986.2 | 35080413 | 35089898 | XM_019351928.1 | XM_019351928.1 PREDICTED: Oreochromis niloticus semaphorin 6B (sema6b), transcript variant X5, mRNA | 1 | 9516 | 9516 | |
| NC_031986.2 | 12928478 | 12928815 | XM_019352179.1 | XM_019352179.1 PREDICTED: Oreochromis niloticus probable uridine nucleosidase 1 (LOC100704461), transcript variant X2, mRNA | 1 | 368 | 368 | |
| NC_031986.2 | 11175537 | 11179966 | XM_005450040.3 | XM_005450040.3 PREDICTED: Oreochromis niloticus amyloid beta precursor protein (app), transcript variant X2, mRNA | 1 | 4460 | 4460 | |
| NC_031984.2 | 22067641 | 22068248 | XM_005478204.3 | XM_005478204.3 PREDICTED: Oreochromis niloticus calsyntenin 1 (clstn1), transcript variant X3, mRNA | 1 | 638 | 638 | |
| NC_031984.2 | 15224199 | 15225087 | XM_014336629.1 | XM_014336629.1 PREDICTED: Haplochromis burtoni glutamate receptor-interacting protein 2-like (LOC102303775), transcript variant X6, mRNA | 1 | 919 | 919 | |
| NC_031982.2 | 27199427 | 27203702 | XM_019348045.1 | XM_019348045.1 PREDICTED: Oreochromis niloticus supervillin (LOC100703985), transcript variant X11, mRNA | 1 | 4306 | 4306 | |
| NC_031982.2 | 1584077 | 1587668 | XR_267431.3 | XR_267431.3 PREDICTED: Oreochromis niloticus junctophilin-1 (LOC100706964), transcript variant X2, misc_RNA | 1 | 3620 | 3620 | |
| NC_031981.2 | 9980440 | 9981823 | XM_006781144.1 | XM_006781144.1 PREDICTED: Neolamprologus brichardi round spermatid basic protein 1-like protein-like (LOC102777401), mRNA | 1 | 1412 | 1412 | |
| NC_031981.2 | 8491949 | 8493661 | XM_019347020.1 | XM_019347020.1 PREDICTED: Oreochromis niloticus leucine-rich repeat-containing protein 17 (LOC100710258), transcript variant X4, mRNA | 1 | 1741 | 1741 | |
| NC_031981.2 | 12545456 | 12549945 | XM_019347073.1 | XM_019347073.1 PREDICTED: Oreochromis niloticus voltage-dependent calcium channel subunit alpha-2/delta-1 (LOC100705144), transcript variant X5, mRNA | 1 | 4520 | 4520 | |
| NC_031981.2 | 10272056 | 10281829 | XR_001224370.2 | XR_001224370.2 PREDICTED: Oreochromis niloticus ELKS/Rab6-interacting/CAST family member 1 (LOC100690550), transcript variant X10, misc_RNA | 1 | 9804 | 9804 | |
| NC_031980.2 | 896114 | 897054 | XR_002058847.1 | XR_002058847.1 PREDICTED: Oreochromis niloticus CDC42 binding protein kinase alpha (cdc42bpa), transcript variant X2, misc_RNA | 1 | 967 | 967 | |
| NC_031980.2 | 32787027 | 32789391 | XM_019346232.1 | XM_019346232.1 PREDICTED: Oreochromis niloticus transmembrane protein 38A (tmem38a), transcript variant X2, mRNA | 1 | 2395 | 2395 | |
| NC_031979.2 | 36408021 | 36408711 | XM_012915678.2 | XM_012915678.2 PREDICTED: Maylandia zebra actinin, alpha 4 (actn4), transcript variant X7, mRNA | 1 | 721 | 721 | |
| NC_031979.2 | 24606047 | 24609702 | XM_005459298.3 | XM_005459298.3 PREDICTED: Oreochromis niloticus membrane metalloendopeptidase (mme), transcript variant X4, mRNA | 1 | 3686 | 3686 | |
| NC_031978.2 | 30702661 | 30704116 | XM_005461429.3 | XM_005461429.3 PREDICTED: Oreochromis niloticus piezo type mechanosensitive ion channel component 1 (piezo1), mRNA | 1 | 1486 | 1486 | |
| NC_031978.2 | 102602 | 105225 | AB270897.1 | AB270897.1 Oreochromis niloticus MHC class IA antigen UBA1, UBA2, UAA1 genes, partial cds, UAA3 and UAA2 pseudogenes, UAA4, UAA5 and UAA6 pseudogene fragments | 1 | 2650 | 2650 | |
| NC_031977.2 | 30895287 | 30896142 | XM_019365760.1 | XM_019365760.1 PREDICTED: Oreochromis niloticus calcium/calmodulin-dependent protein kinase type II subunit beta (LOC100695100), transcript variant X32, mRNA | 1 | 886 | 886 | |
| NC_031977.2 | 19508827 | 19509068 | XM_019365292.1 | XM_019365292.1 PREDICTED: Oreochromis niloticus ubiquitin-associated protein 2 (LOC100696291), transcript variant X4, mRNA | 1 | 272 | 272 | |
| NC_031976.2 | 4608763 | 4618143 | XM_019358987.1 | XM_019358987.1 PREDICTED: Oreochromis niloticus RNA-directed DNA polymerase from mobile element jockey-like (LOC109202218), partial mRNA | 1 | 9409 | 9409 | |
| NC_031976.2 | 36016595 | 36018688 | XM_013275037.2 | XM_013275037.2 PREDICTED: Oreochromis niloticus lamin A/C (lmna), transcript variant X2, mRNA | 1 | 2124 | 2124 | |
| NC_031973.2 | 9903733 | 9905025 | XM_019362446.1 | XM_019362446.1 PREDICTED: Oreochromis niloticus eukaryotic elongation factor 2 kinase (eef2k), transcript variant X7, mRNA | 1 | 1321 | 1321 | |
| NC_031973.2 | 6541346 | 6542303 | XM_019362294.1 | XM_019362294.1 PREDICTED: Oreochromis niloticus ankyrin-3 (LOC100703272), transcript variant X11, mRNA | 1 | 986 | 986 | |
| NC_031973.2 | 21793231 | 21795462 | XR_002063228.1 | XR_002063228.1 PREDICTED: Oreochromis niloticus sorting nexin 29 (snx29), transcript variant X2, misc_RNA | 1 | 2262 | 2262 | |
| NC_031972.2 | 9033630 | 9035959 | XM_019360763.1 | XM_019360763.1 PREDICTED: Oreochromis niloticus myocyte-specific enhancer factor 2C (LOC100706088), transcript variant X4, mRNA | 1 | 2358 | 2358 | |
| NC_031972.2 | 61245143 | 61245486 | XM_019361891.1 | XM_019361891.1 PREDICTED: Oreochromis niloticus microtubule associated monooxygenase, calponin and LIM domain containing 3 (mical3), transcript variant X3, mRNA | 1 | 374 | 374 | |
| NC_031972.2 | 48411676 | 48413963 | XM_014335746.1 | XM_014335746.1 PREDICTED: Haplochromis burtoni CUGBP Elav-like family member 2 (LOC102310863), mRNA | 1 | 2318 | 2318 | |
| NC_031972.2 | 41189270 | 41189586 | XM_019357934.1 | XM_019357934.1 PREDICTED: Oreochromis niloticus suppression of tumorigenicity 7 (st7), transcript variant X1, mRNA | 1 | 347 | 347 | |
| NC_031972.2 | 3583995 | 3584602 | XM_005449241.2 | XM_005449241.2 PREDICTED: Oreochromis niloticus troponin T3, fast skeletal type (tnnt3), transcript variant X6, mRNA | 1 | 636 | 636 | |
| NC_031971.2 | 42021773 | 42028544 | AB270897.1 | AB270897.1 Oreochromis niloticus MHC class IA antigen UBA1, UBA2, UAA1 genes, partial cds, UAA3 and UAA2 pseudogenes, UAA4, UAA5 and UAA6 pseudogene fragments | 1 | 6802 | 6802 | |
| NC_031971.2 | 27059186 | 27061091 | XM_006787490.1 | XM_006787490.1 PREDICTED: Neolamprologus brichardi ubinuclein-2-like (LOC102799674), mRNA | 1 | 1936 | 1936 | |
| NC_031971.2 | 24932987 | 24933518 | XM_013272974.2 | XM_013272974.2 PREDICTED: Oreochromis niloticus nuclear factor 1 X-type (LOC100690838), transcript variant X14, mRNA | 1 | 562 | 562 | |
| NC_031970.2 | 32666106 | 32668191 | XM_005450143.3 | XM_005450143.3 PREDICTED: Oreochromis niloticus PDZ domain containing ring finger 3 (pdzrn3), transcript variant X2, mRNA | 1 | 2116 | 2116 | |
| NC_031969.2 | 35390737 | 35394011 | XM_019357502.1 | XM_019357502.1 PREDICTED: Oreochromis niloticus cell division cycle 27 (cdc27), transcript variant X2, mRNA | 1 | 3305 | 3305 | |
| NC_031967.2 | 32889860 | 32890096 | XM_019353193.1 | XM_019353193.1 PREDICTED: Oreochromis niloticus NHS like 2 (nhsl2), transcript variant X5, mRNA | 1 | 267 | 267 | |
| NC_031966.2 | 31950713 | 31953718 | XM_013264433.2 | XM_013264433.2 PREDICTED: Oreochromis niloticus zinc finger DHHC-type containing 2 (zdhhc2), transcript variant X3, mRNA | 1 | 3036 | 3036 | |
| NC_031966.2 | 25348411 | 25349729 | XM_012924841.2 | XM_012924841.2 PREDICTED: Maylandia zebra myozenin-2-like (LOC101481626), mRNA | 1 | 1349 | 1349 | |
| NC_031966.2 | 21354417 | 21356032 | XM_006781341.1 | XM_006781341.1 PREDICTED: Neolamprologus brichardi probable E3 ubiquitin-protein ligase MID2-like (LOC102785246), transcript variant X5, mRNA | 1 | 1646 | 1646 | |
| NC_031965.2 | 7604686 | 7609175 | XM_019346135.1 | XM_019346135.1 PREDICTED: Oreochromis niloticus neuron navigator 2 (LOC100704374), mRNA | 1 | 4518 | 4518 | |
| NC_031965.2 | 19619094 | 19619304 | XR_002061999.1 | XR_002061999.1 PREDICTED: Oreochromis niloticus transcription factor SOX-6 (LOC100694759), transcript variant X4, misc_RNA | 1 | 241 | 241 | |
| Gene ID | Gene coordinates start | Gene coordinates end | Gene of origin for circRNAs ID | Gene of origin for circRNAs | Number of circRNAs | Minimum size, bp | Maximum size, bp | |
| NC_031987.2 | 21667160 | 21673355 | AB270897.1 | AB270897.1 Oreochromis niloticus MHC class IA antigen UBA1, UBA2, UAA1 genes, partial cds, UAA3 and UAA2 pseudogenes, UAA4, UAA5 and UAA6 pseudogene fragments | 1 | 6226 | 6226 | |
| NC_031986.2 | 45119249 | 45119970 | XM_013268343.2 | XM_013268343.2 PREDICTED: Oreochromis niloticus nexilin F-actin binding protein (nexn), transcript variant X3, mRNA | 1 | 752 | 752 | |
| NC_031986.2 | 12928478 | 12928815 | XM_019352179.1 | XM_019352179.1 PREDICTED: Oreochromis niloticus probable uridine nucleosidase 1 (LOC100704461), transcript variant X2, mRNA | 1 | 368 | 368 | |
| NC_031984.2 | 22067641 | 22068248 | XM_005478204.3 | XM_005478204.3 PREDICTED: Oreochromis niloticus calsyntenin 1 (clstn1), transcript variant X3, mRNA | 1 | 638 | 638 | |
| NC_031984.2 | 15224199 | 15225087 | XM_014336629.1 | XM_014336629.1 PREDICTED: Haplochromis burtoni glutamate receptor-interacting protein 2-like (LOC102303775), transcript variant X6, mRNA | 1 | 919 | 919 | |
| NC_031982.2 | 27199427 | 27203702 | XM_019348045.1 | XM_019348045.1 PREDICTED: Oreochromis niloticus supervillin (LOC100703985), transcript variant X11, mRNA | 1 | 4306 | 4306 | |
| NC_031982.2 | 23105201 | 23105671 | XM_003448490.3 | XM_003448490.3 PREDICTED: Oreochromis niloticus complexin-4 (LOC100705335), transcript variant X1, mRNA | 1 | 501 | 501 | |
| NC_031982.2 | 22933984 | 22934532 | XM_005476595.3 | XM_005476595.3 PREDICTED: Oreochromis niloticus insulin receptor (LOC100696191), mRNA | 1 | 579 | 579 | |
| NC_031982.2 | 19077768 | 19078862 | XM_019347895.1 | XM_019347895.1 PREDICTED: Oreochromis niloticus obscurin, cytoskeletal calmodulin and titin-interacting RhoGEF (obscn), transcript variant X2, mRNA | 1 | 1125 | 1125 | |
| NC_031982.2 | 13444988 | 13479806 | XM_005476190.3 | XM_005476190.3 PREDICTED: Oreochromis niloticus myomegalin (LOC100703940), transcript variant X7, mRNA | 1 | 34849 | 34849 | |
| NC_031981.2 | 10238150 | 10281829 | XM_005451686.3 | XM_005451686.3 PREDICTED: Oreochromis niloticus trichohyalin-like (LOC102077753), mRNA | 1 | 43710 | 43710 | |
| NC_031979.2 | 35013862 | 35016025 | XM_013913810.1 | XM_013913810.1 PREDICTED: Pundamilia nyererei hypoxia-inducible factor 1-alpha-like (LOC102195817), mRNA | 1 | 2194 | 2194 | |
| NC_031979.2 | 24606047 | 24609702 | XM_005459298.3 | XM_005459298.3 PREDICTED: Oreochromis niloticus membrane metalloendopeptidase (mme), transcript variant X4, mRNA | 1 | 3686 | 3686 | |
| NC_031978.2 | 30702661 | 30704116 | XM_005461429.3 | XM_005461429.3 PREDICTED: Oreochromis niloticus piezo type mechanosensitive ion channel component 1 (piezo1), mRNA | 1 | 1486 | 1486 | |
| NC_031978.2 | 102602 | 105225 | AB270897.1 | AB270897.1 Oreochromis niloticus MHC class IA antigen UBA1, UBA2, UAA1 genes, partial cds, UAA3 and UAA2 pseudogenes, UAA4, UAA5 and UAA6 pseudogene fragments | 1 | 2650 | 2650 | |
| NC_031977.2 | 20038517 | 20040903 | XR_266173.3 | XR_266173.3 PREDICTED: Oreochromis niloticus sperm tail PG-rich repeat containing 2 (stpg2), transcript variant X3, misc_RNA | 1 | 2417 | 2417 | |
| NC_031977.2 | 19508827 | 19509068 | XM_019365292.1 | XM_019365292.1 PREDICTED: Oreochromis niloticus ubiquitin-associated protein 2 (LOC100696291), transcript variant X4, mRNA | 1 | 272 | 272 | |
| NC_031976.2 | 36016595 | 36018688 | XM_013275037.2 | XM_013275037.2 PREDICTED: Oreochromis niloticus lamin A/C (lmna), transcript variant X2, mRNA | 1 | 2124 | 2124 | |
| NC_031975.2 | 4684415 | 4687685 | XM_005454560.3 | XM_005454560.3 PREDICTED: Oreochromis niloticus PBX/knotted 1 homeobox 2 (pknox2), transcript variant X1, mRNA | 1 | 3299 | 3299 | |
| NC_031973.2 | 9903733 | 9905025 | XM_019362446.1 | XM_019362446.1 PREDICTED: Oreochromis niloticus eukaryotic elongation factor 2 kinase (eef2k), transcript variant X7, mRNA | 1 | 1321 | 1321 | |
| NC_031972.2 | 9033630 | 9035959 | XM_019360763.1 | XM_019360763.1 PREDICTED: Oreochromis niloticus myocyte-specific enhancer factor 2C (LOC100706088), transcript variant X4, mRNA | 1 | 2358 | 2358 | |
| NC_031972.2 | 48409310 | 48413963 | XM_014335746.1 | XM_014335746.1 PREDICTED: Haplochromis burtoni CUGBP Elav-like family member 2 (LOC102310863), mRNA | 1 | 4684 | 4684 | |
| NC_031972.2 | 3588635 | 3589170 | XM_005449247.3 | XM_005449247.3 PREDICTED: Oreochromis niloticus troponin T3, fast skeletal type (tnnt3), transcript variant X12, mRNA | 1 | 564 | 564 | |
| NC_031971.2 | 42021773 | 42028544 | AB270897.1 | AB270897.1 Oreochromis niloticus MHC class IA antigen UBA1, UBA2, UAA1 genes, partial cds, UAA3 and UAA2 pseudogenes, UAA4, UAA5 and UAA6 pseudogene fragments | 1 | 6802 | 6802 | |
| NC_031971.2 | 4075465 | 4076159 | XM_005461334.3 | XM_005461334.3 PREDICTED: Oreochromis niloticus endoplasmic reticulum oxidoreductase 1 beta (ero1b), transcript variant X2, mRNA | 1 | 723 | 723 | |
| NC_031971.2 | 27059186 | 27061091 | XM_006787490.1 | XM_006787490.1 PREDICTED: Neolamprologus brichardi ubinuclein-2-like (LOC102799674), mRNA | 1 | 1936 | 1936 | |
| NC_031970.2 | 24263108 | 24264783 | XM_019358977.1 | XM_019358977.1 PREDICTED: Oreochromis niloticus gastrula zinc finger protein XlCGF57.1 (LOC100709046), transcript variant X26, mRNA | 1 | 1706 | 1706 | |
| NC_031967.2 | 3188565 | 3190679 | XM_019349016.1 | XM_019349016.1 PREDICTED: Oreochromis niloticus echinoderm microtubule associated protein like 3 (eml3), transcript variant X2, mRNA | 1 | 2143 | 2143 | |
